# Modelling prognostic trajectories of cognitive decline due to Alzheimer's disease

**DOI:** 10.1016/j.nicl.2020.102199

**Published:** 2020-01-26

**Authors:** Joseph Giorgio, Susan M. Landau, William J. Jagust, Peter Tino, Zoe Kourtzi, for the Alzheimer's Disease Neuroimaging Initiative

**Affiliations:** a Department of Psychology, University of Cambridge, Cambridge, United Kingdom; b Helen Wills Neuroscience Institute, University of California, Berkeley, CA USA; c School of Computer Science, University of Birmingham, Birmingham, United Kingdom

**Keywords:** Machine learning, Mild cognitive impairment, Alzheimer's disease brain imaging, Cognition

## Abstract

Alzheimer's disease (AD) is characterised by a dynamic process of neurocognitive changes from normal cognition to mild cognitive impairment (MCI) and progression to dementia. However, not all individuals with MCI develop dementia. Predicting whether individuals with MCI will decline (i.e. progressive MCI) or remain stable (i.e. stable MCI) is impeded by patient heterogeneity due to comorbidities that may lead to MCI diagnosis without progression to AD. Despite the importance of early diagnosis of AD for prognosis and personalised interventions, we still lack robust tools for predicting individual progression to dementia. Here, we propose a novel trajectory modelling approach based on metric learning (Generalised Metric Learning Vector Quantization) that mines multimodal data from MCI patients in the Alzheimer's disease Neuroimaging Initiative (ADNI) cohort to derive individualised prognostic scores of cognitive decline due to AD. We develop an integrated biomarker generation– using partial least squares regression– and classification methodology that extends beyond binary patient classification into discrete subgroups (i.e. stable vs. progressive MCI), determines individual profiles from baseline (i.e. cognitive or biological) data and predicts individual cognitive trajectories (i.e. change in memory scores from baseline). We demonstrate that a metric learning model trained on baseline cognitive data (memory, executive function, affective measurements) discriminates stable vs. progressive MCI individuals with high accuracy (81.4%), revealing an interaction between cognitive (memory, executive functions) and affective scores that may relate to MCI comorbidity (e.g. affective disturbance). Training the model to perform the same binary classification on biological data (mean cortical β-amyloid burden, grey matter density, APOE 4) results in similar prediction accuracy (81.9%). Extending beyond binary classifications, we develop and implement a trajectory modelling approach that shows significantly better performance in predicting individualised rate of future cognitive decline (i.e. change in memory scores from baseline), when the metric learning model is trained with biological (*r* = −0.68) compared to cognitive (*r* = −0.4) data. Our trajectory modelling approach reveals interpretable and interoperable markers of progression to AD and has strong potential to guide effective stratification of individuals based on prognostic disease trajectories, reducing MCI patient misclassification, that is critical for clinical practice and discovery of personalised interventions.

## Highlights

• Metric learning reveals continuous prognostic scores of cognitive decline due to AD.
• Individualised disease trajectory modelling benefits from adding non-invasive biomarkers.
• Interpretable and interoperable markers of progression to dementia for patient stratification.

## 1 Introduction

Progression to dementia due to Alzheimer's Disease (AD) involves multiple pathways of disease pathophysiology that impact cognition (Jack et al., 2018, 2013; Jagust, 2018). Individuals who develop dementia follow a trajectory from a stage of normal cognition to Mild Cognitive Impairment (MCI) and subsequent dementia (McKhann et al., 2011; Petersen et al., 2001; Sperling et al., 2011). Predicting early onset of neurocognitive decline due to AD has major implications for timely clinical management and patient outcomes. Yet, diagnosis at early stages of disease is impeded by heterogeneity in patient populations due to comorbidities (e.g. affective or cerebrovascular disorders) that may lead to MCI diagnosis without further progression to AD (Petersen, 2009). Determining disease trajectories for individuals diagnosed with MCI has major implications for prognosis and personalised interventions.

Recent advances in machine learning allow us to develop predictive models of neurodegenerative disease by mining multimodal datasets that include measurements of cognition and neuropathology from large patient cohorts (Woo et al., 2017). In line with the 2011 NIA-AA diagnostic framework for mild cognitive impairment or dementia stages in AD (Albert et al., 2011; McKhann et al., 2011), most machine learning models in AD have focused on binary classifications. For example, machine learning models have been shown to predict with high accuracy whether individuals diagnosed with MCI will decline (i.e. progressive MCI; pMCI) or remain stable (i.e. stable MCI; sMCI) (Rathore et al., 2017). Fewer models have achieved prediction of individual variability in disease progression (Tang et al., 2015; Woo et al., 2017) focusing primarily on probabilistic estimates of time to conversion to AD (Alsaedi et al., 2018; Casanova et al., 2013; Desikan et al., 2010; Jack et al., 2010; Liu et al., 2017; Michaud et al., 2017; Oulhaj et al., 2009; Young et al., 2014), with some models estimating exact time to conversion (Dukart et al., 2015; Thung et al., 2018; Vogel et al., 2018).

Despite the high prediction accuracies achieved by machine learning algorithms, binary classification approaches are poorly constrained, as they are based on clinical labels rather than capturing information in longitudinal patient trajectories. As a result, individual patients at the class boundary that differ only slightly in their profile may be misclassified. Further, the validity and statistical power (Li et al., 2019) of these approaches is limited by the frequency of clinical follow-ups (i.e. the point of conversion may occur between clinical assessments) and inter-rater reliability (i.e. clinicians may differ in their assessment). Extending machine learning modelling to predict measures determined by diagnostic labelling (i.e. time to conversion) suffers from the same limitations, introducing bias and limiting the interpretability and interoperability of machine learning algorithms (Janssen et al., 2018). Thus, novel modelling approaches that predict individualised trajectories of cognitive decline based on continuous measures need to be developed to enhance clinical validity and guide effective clinical interventions and drug discovery trials.

Here, we develop and implement a trajectory modelling approach that extends beyond binary classification. We use machine learning (metric learning) algorithms to stratify patients at early stages of impairment (i.e. MCI) based on baseline cognitive or biological data and determine individual prognostic trajectories based on continuous measures of cognitive decline (i.e. change in memory scores over time). Our trajectory modelling approach allows us to extract continuous information about progression to AD, in line with the current 2018 NIA-AA research framework that has transitioned to defining AD as a continuum (Jack et al., 2018).

In particular, we used large-scale data from the Alzheimer's disease Neuroimaging Initiative (ADNI) database. Cognitive data comprise composite scores across tasks; that is, summative measures of memory (i.e., ADNI-Mem (Crane et al., 2012)), executive function (i.e., ADNI–EF (Gibbons et al., 2012)), and depression (Yesavage, 1988). Similar composite measures have been shown to be effective for diagnosing cognitive dysfunction (Ayutyanont et al., 2014; Donohue et al., 2014; Jutten et al., 2018; 2017; Langbaum et al., 2014). In addition, we used well-studied biomarkers of AD (Jagust, 2018; Resnick and Sojkova, 2011); that is, grey matter density derived from structural MRI scans, β-amyloid burden from PET scans and APOE 4 status.

**Fig. 1:**
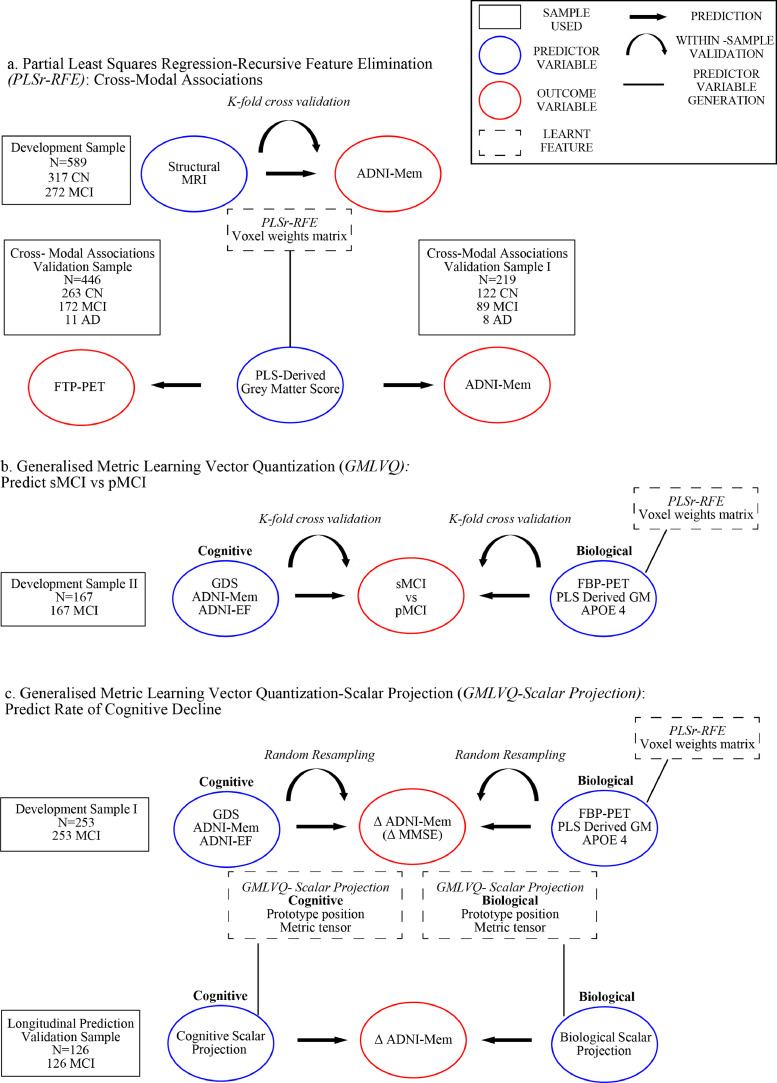
Modelling framework. **a**. PLSr-RFE was used to generate the PLS derived grey matter score and out-of-sample-tests for cross modal associations. Using the Development Sample a voxel weights matrix is learned and validated using k-fold cross-validation to predict ADNI-Mem. Using this voxel weights matrix, we generated the PLS derived grey matter score for the Cross-modal associations validation sample to test the cross modal association between the PLS derived grey matter score and cortical tau (flortaucipir, or FTP-PET). We used the Cross-modal Associations validation sample I to out-of-sample validate the relationship between the PLS derived grey matter score and ADNI-Mem. The voxel weights matrix generated is then used to derive the PLS derived grey matter feature for the data used in panels b and c. **b.** GMLVQ binary classification was used to discriminate between sMCI and pMCI based on biological or cognitive data. Using Development sample II, GMLVQ binary classifiers were trained and validated using k-fold cross-validation to predict progression to dementia from MCI (sMCI vs pMCI). (**c)** GMLVQ-Scalar Projection was used to generate the cognitive and biological scalar projections and out-of-sample validate the relationship of the scalar projections with rate of future cognitive decline. Using Development Sample I, cognitive and biological scalar projections were generated and correlated to rate of change in future ADNI-Mem scores. This relationship was validated using random resampling. Further, the relationship of the scalar projection and rate of future cognitive decline was validated with rate of change of future MMSE using Development Sample I. The prototype position and metric tensor learned from the GMLVQ-Scalar projection using Development Sample I were then used to derive the cognitive and biological scalar projections for the longitudinal prediction validation sample to test the out-of-sample relationship between the scalar projection and rate of future cognitive decline.

We adopted a metric learning framework (Generalised Metric Learning Vector Quantization, GMLVQ) and extended our approach beyond binary classification (i.e. sMCI vs. pMCI) to modelling of continuous measurements (i.e. change in ADNI-Mem scores) (Figure 1). In particular, we first tested a low-parameter, interpretable model on a binary classification task (sMCI vs. pMCI) and interrogated the key cognitive predictors that separate sMCI vs. pMCI individuals. This modelling revealed ADNI-Mem as the most discriminative cognitive feature for classifying sMCI vs. pMCI, in line with previous work showing that ADNI-Mem captures memory performance in amnestic MCI populations (Crane et al., 2012). We then developed a novel feature selection and construction method based on partial least squares regression (PLSr) to generate an interpretable and interoperable disease-specific biomarker (i.e. grey matter atrophy due to AD) that predicts memory deficits as measured by ADNI-Mem, discriminates sMCI vs. pMCI individuals and relates to individual tau burden, as measured by flortaucipir PET in an independent sample. We then trained our metric learning model on biological data– including the PLS-derived grey-matter feature, mean cortical β-amyloid burden, and APOE 4– and compared the classification accuracy across models trained with either cognitive or biological data.

To extend our modelling approach beyond binary classification (i.e. sMCI vs. pMCI), we derived a scalar projection (i.e. distance of each individual from the sMCI prototype) based on the metric learning model that allows us to determine a continuous metric of disease progression. We demonstrate that this metric relates to rate of future cognitive decline (i.e. change in ADNI-Mem scores following baseline), providing evidence that our methodology delivers a continuous prognostic score of individual cognitive decline due to AD. Further, our trajectory modelling approach determines predictive cognitive markers of individual variability in AD progression; yet, predicting disease trajectories improves when including non-invasively measured and interpretable biomarkers (i.e. grey matter density and/or APOE 4).

## 2 Methods and materials

### 2.1 ADNI participants

Data were obtained from the Alzheimer's disease Neuroimaging Initiative (ADNI) database (adni.loni.usc.edu). ADNI was launched in 2003 as a public-private partnership, led by Principal Investigator Michael W. Weiner, MD. A major goal of ADNI has been to examine biomarkers including serial magnetic resonance imaging (MRI), and positron emission tomography (PET), with clinical and neuropsychological assessment to predict outcomes in mild cognitive impairment (MCI) and early Alzheimer's disease (AD). Data samples are defined as: (1) Development data samples used for model formulation and within-sample validation, (2) independent validation data samples used for out-of-sample validation. Below we provide details for each data sample:

#### 2.1.1 Development sample

Data from 589 individuals (baseline diagnoses: Normal =317, MCI=272) from ADNI-GO and ADNI-2 were used for model formulation and within-sample validation. For these individuals the baseline assessments (MRI and cognitive) were those closest to the time of the first florbetapir (FBP) PET scan. All individuals had baseline cognitive measurements, 3T structural MRI, FBP-PET scan for measuring β-amyloid, and APOE genotyping. All individuals were included in this cross sectional sample independent of their future diagnosis (i.e. whether following baseline an individual's diagnosis changed from cognitively normal to MCI/AD).

#### 2.1.2 Development sample I

253 MCI individuals (of a total of 272) have at least 3 longitudinal cognitive testing sessions. Data from these individuals were used for model formulation and within-sample validation for the continuous longitudinal outcome prediction models.

#### 2.1.3 Development sample II

167 MCI individuals (of the 253 MCI individuals in Development Sample I) have 3 years of clinical diagnostic assessments. Data from these individuals were used as dichotomous outcomes (stable vs. progressive MCI) for longitudinal predictions.

#### 2.1.4 Cross-modal associations validation sample

To out-of-sample-validate the model that predicted cross-modality associations (e.g. predict ADNI-Mem scores from grey-matter), we drew an independent validation sample comprised of 446 individuals (Normal=263, MCI=172, AD=11) from ADNI-3. These individuals have a 3T structural MRI, and cognitive measures in addition to a flortaucipir (FTP) PET scan for measuring cortical tau.

#### 2.1.5 Cross-modal associations validation sample I

We selected 219 individuals from the Cross-modal associations validation sample (Normal=122, MCI=89, AD=8), excluding individuals with an FTP-PET scan who were part of the Development Sample. Individuals in the Cross-modal associations validation sample I were newly recruited into ADNI-3, that is they had not been enroled in ADNI-GO or ADNI-2 prior to enroling in ADNI-3. This independent sample was used to validate cross-modal associations of grey matter and ADNI-Mem scores. All data from the Cross-modal associations validation sample were taken from assessments closest in time to the FTP-PET scan.

#### 2.1.6 Longitudinal prediction validation sample

To out-of-sample-validate the model that generated longitudinal predictions, we drew an independent validation sample comprising 126 MCI individuals (ADNI-GO, ADNI-2). These individuals have baseline cognitive, 3T structural MRI, FBP-PET measurements and APOE 4 genotyping. As for the data used for model formulation, baseline was defined as the assessment closest in time to an individual's first FBP-PET scan acquired in ADNI. These individuals also have at least 3 longitudinal cognitive testing sessions that were used to validate the outcome measures for longitudinal predictions. See Supplementary *Table S1* for sample demographics.

## 3 Brain imaging data

### 3.1 MRI acquisition

Structural MRIs were acquired at ADNI-GO, ADNI-2 and ADNI-3 sites equipped with 3 T MRI scanners using a 3D MP-RAGE or IR-SPGR T1-weighted sequences, as described online (http://adni.loni.usc.edu/methods/documents/mri-protocols).

### 3.2 PET acquisition

PET imaging was performed at each ADNI site according to standardised protocols. The FBP-PET protocol entailed the injection of 10 mCi with acquisition of 20 min of emission data at 50–70 min post injection. The FTP-PET protocol entailed the injection of 10 mCi of tracer followed by acquisition of 30 min of emission data from 75–105 min post injection.

### 3.3 Image analysis: FTP (Flortaucipir PET) Tau

FTP data were realigned, and the mean of all frames was used to coregister FTP to each participant's MRI acquired closest to the time of the FTP-PET. FTP standardised uptake value ratio (SUVR) images were normalised to inferior cerebellar grey matter (Baker et al., 2017). MR images were segmented and parcellated using Freesurfer (V5.3) and regions of interest were used to extract cerebellar-normalised regional SUVR data. SUVR data was summarised for three Braak staging regions 12 (medial temporal), 34 (inferolateral temporal) and 56 (extra-temporal neocortical) by averaging uptake across individual Freesurfer region of interests (ROIs) comprising each Braak region (Maass et al., 2017). Finally, we assigned individuals as tau positive for each Braak stage if their SUVR value was greater than the 90th percentile of amyloid-negative, cognitively normal individuals.

### 3.4 Image analysis: FBP (Florbetapir PET) beta amyloid

FBP data were realigned, and the mean of all frames was used to co-register FBP data to each participant's structural MRI. Cortical SUVRs were generated by averaging FBP retention in a standard group of ROIs (lateral and medial frontal, anterior and posterior cingulate, lateral parietal, and lateral temporal cortical grey matter) and dividing by the average uptake from a composite reference region (including the whole cerebellum, pons/brainstem, and eroded subcortical white matter regions) to create an index of global cortical FBP burden for each subject (Landau et al., 2015).

### 3.5 Image analysis voxel based morphometry (VBM)

Structural scans were segmented into grey matter, white matter and CSF (Cerebrospinal Fluid). The DARTEL toolbox (Ashburner, 2007) was then used to generate a study specific template to which all scans were normalised. Following this, individual grey matter segmentation volumes were normalised to MNI space without modulation. The unmodulated values for each voxel represent grey matter density at the voxel location. We chose to use the unmodulated grey matter data as it has been shown that there is a marked decrease in sensitivity to detecting abnormal regions within grey matter when the data is modulated (Radua et al., 2014) (for analysis with modulated data, see Supplementary *Figure S4b*, Supplementary *Table S2b*).

All images were then smoothed using a 3 mm3 isotropic kernel and resliced to MNI resolution 1.5 × 1.5 × 1.5 mm voxel size. We used a small kernel size, as topographically complex and relatively small cortical regions are likely to be affected in AD (i.e. structures within the medial temporal cortex; e.g. hippocampus, entorhinal cortex). It has been suggested that smoothing beyond a 3 mm kernel may artificially link small but discrete clusters of voxels, reducing topographic sensitivity (Radua et al., 2014). Further, our analysis applies a spatial decomposition across voxels. By sampling the spatial covariance structure across voxels, disease related non-parametric variations at the voxel level (that are mitigated using larger smoothing kernels in parametric statistical tests across participants) are preserved when using smaller kernel sizes, improving the efficacy of the analysis method. All structural MRI pre-processing was performed using Statistical Parametric Mapping 12 (http://www.fil.ion.ucl.ac.uk/spm/).

### 3.6 Cognitive scores

We used three baseline cognitive scores as predictors for longitudinal models: a) composite scores of memory function (ADNI-Mem) derived from the Rey Auditory Verbal Learning, AD Assessment Schedule-Cognition, Mini-Mental State Examination and Logical Memory tests (Crane et al., 2012). b) composite scores of executive function (ADNI-EF) derived from the WAIS-R Digit Symbol Substitution, Digit Span Backwards, Trails A and B, Category Fluency and Clock Drawing tests (Gibbons et al., 2012). c) the sum of all elements from the geriatric depression scale (GDS) (Yesavage, 1988). As individuals are excluded from ADNI with a GDS >5 we investigate affective disturbance at subthreshold levels of clinical depression.

### 3.7 Generalised Metric Learning Vector Quantization (GMLVQ)

We used the Generalised Metric Learning Vector Quantization (GMLVQ) framework (Schneider et al., 2009) to generate and test binary classification models (Supplementary *Methods GMLVQ)* that classify sMCI vs. pMCI individuals (Development Sample II). Individuals were characterised as sMCI if they repeatedly received an MCI diagnosis for more than three years of clinical observation. Individuals who progressed from MCI to AD within a window of 3 years of clinical observation were characterised as pMCI. Individuals who progressed from MCI to AD after 3 years were excluded from the Development Sample II.

GMLVQ belongs to the class of classifiers referred to as Learning Vector Quantization (LVQ). These classifiers operate in a supervised manner to iteratively modify class-specific prototypes and learn boundaries between classes. For each training example, the closest prototype of each class is determined, these prototypes are then updated so that the prototype defining the same class is moved towards the training example and other prototype(s) representing different class(es) are moved further away. The Generalised Metric LVQ (GMLVQ) extends the LVQ utilising a full metric-tensor for a more robust distance measure. By applying the metric-tensor, specific feature scaling can occur while also accounting for different feature scales and pairwise task-conditional dependencies in the input space. Interrogating the diagonal terms allow us to determine the key univariate predictors for separating sMCI vs. pMCI patients. Further, interrogating the off diagonal terms of the metric tensor allow us to investigate the multivariate predictors that contribute to this classification task.

### 3.8 GMLVQ cognitive model

We used the (GMLVQ) framework to generate and test binary classification models that classify sMCI vs. pMCI individuals (Development Sample II) based on cognitive measures (GDS, ADNI-Mem and ADNI-EF).

### 3.9 Partial least squares regression with recursive feature elimination (PLSr-RFE)

We implemented Partial Least Squares Regression with Recursive Feature Elimination (PLSr-RFE) (Supplementary *Methods PLS)* to generate a grey matter density feature based on data from the Development sample (normal and MCI individuals)*.* All 3T structural MRI scans in the Development sample were collected using a 3D MP-RAGE T1-weighted sequence. In particular, we used grey matter density measured by structural MRI as a predictor variable to determine multivariate relationships between grey matter voxels that best predict ADNI-Mem, as our GMLVQ modelling showed ADNI-Mem to be the most heavily weighted cognitive feature for the sMCI vs. pMCI classification. (Supplementary *Methods PLS)*. We performed feature set construction using PLSr and feature reduction using recursive feature elimination. PLSr determines multivariate relationships between predictor variables to best describe response variables. In particular, PLSr applies a decomposition on a set of predictors to create orthogonal latent variables that show the maximum covariance with the response variables (Krishnan et al., 2011; McIntosh and Lobaugh, 2004). In our study, we used PLSr to generate a set of latent predictor variables from structural MRI data, where a) the number of features (i.e. grey matter voxels) is far greater than the number of observations (e.g. number of voxels >300,000, number of observations <1000), b) there is high degree of multi-collinearity between voxels. PLSr reduces redundant information and maximises the amount of variance that the latent variables predict in the response variable. Further, we performed recursive feature elimination by iteratively removing voxels that have weak predictive value. To determine the optimal number of grey matter voxels to be retained, we used a 5 fold nested cross validation and an early stopping paradigm (Supplementary *Methods, PLSr Recursive Feature Elimination)*.

### 3.10 GMLVQ biological model

We followed the same methodology as for the **GMLVQ Cognitive model** (Development Sample II) to test the GMLVQ model on biological data. That is, we generated and tested binary classification models based on metric learning that discriminate between the same sMCI vs. pMCI individuals based on biological data (PLS derived grey matter score, β-amyloid and APOE 4) (Supplementary *Methods GMLVQ, Figure S1)*. Note that this sample includes 3 pMCI individuals who were β-amyloid negative (i.e. SUVR<1.11) at baseline. We did not restrict our measure of β-amyloid to a binary value but rather used continuous SUVR values to avoid model bias near the ADNI threshold for amyloid positivity.

### 3.11 GMLVQ – scalar projection

We next generated a continuous prediction using either baseline cognitive data (GDS, ADNI-Mem, ADNI-EF) or baseline biological data (PLS Derived Grey matter score, β-amyloid, APOE 4) for MCI individuals (Development sample I). The GMLVQ- Scalar Projection method extends the GMLVQ framework to extract specific distance information from the sample vector *x_i_* and the learnt prototypes *w_(stable,progressive)_*. Specifically, we determine the distance in the learnt space (i.e. after applying the learnt metric tensor) between an individual with sample vector *x_i_* and the learnt prototype *w_stable_* along the vector separating *w_stable_* and *w_progressive_* (Supplementary *Methods GMLVQ – Scalar Projection, Figure S2).*

A value of 1 indicates that a sample vector is incident to the pMCI prototype whereas a value of 0 indicates that a sample vector is incident to the sMCI prototype, and a value of 0.5 is the decision boundary separating the two classes within the binary classification framework. The scalar projection has a large positive value for pMCI individuals and zero or negative value for sMCI individuals (Supplementary *Figure S3*).

### 3.12 Relating the scalar projection to individual rates of future cognitive decline

We used the GMLVQ-Scalar projection method for 253 MCI individuals (Development Sample I) to generate a cognitive scalar projection from baseline cognitive variables (GDS, ADNI-Mem, ADNI-EF), and a biological scalar projection from baseline biological variables (PLS Derived Grey matter score, β-amyloid, APOE 4). To test whether individual scalar projections relate to individual rates of future cognitive decline, we correlated (Pearson's correlation) the scalar projection (generated using baseline predictors) with the rate of future change in ADNI-Mem scores. We computed the rate of future cognitive change by fitting a linear model to the ADNI-Mem scores across multiple measurements (Development Sample I: mean=5.7, std=1 time points; mean=4, std=1.7 years, Longitudinal prediction validation sample: mean=5, std=1.7 time points; mean=4.4, std=1.5 years). The slope of the linear model represents the rate of change in ADNI-Mem score. Individual scores higher than 2 standard deviations from the sMCI mean score or less than 2 standard deviations from the pMCI mean score were determined as outliers and excluded from further analysis.

## 4 Statistical validation

### 4.1 Within-sample validation

To test within-sample generalisability for the GMLVQ (Development Sample II) and PLSr-RFE (Development Sample) models we use k-fold cross validation. Within each cross fold we select hyper-parameters using nested cross-validation. To assess model generalisation performance, we averaged metrics (GMLVQ: Accuracy, Macro Averaged Error (MAE), True Positive (TP), True Negative (TN); PLSr-RFE: Variance Explained) from the test set across all cross folds. Within-sample generalisation for the GMLVQ-scalar projection framework (Development Sample I) was assessed using random resampling (1000 resamplings). We assessed within-sample generalisation based on the median of the correlation coefficients generated from the test sets across resampling using 95% confidence intervals.

### 4.2 Out-of-Sample validation

We test the out-of-sample association of the PLS derived grey matter feature (represented by the voxel weight matrix) with memory (Cross-modal associations validation sample I) and cortical tau (Cross-modal associations validation sample) from the 3 selected Braak regions. Finally, we test the out-of-sample generalisability of the GMLVQ-Scalar Projection in predicting individual rates of future cognitive decline (Longitudinal prediction validation sample). To ensure that our PLS-grey matter feature is robust to different scanner sequences, we included 3T structural MRI scans collected using either a 3D MP-RAGE or IR-SPGR T1-weighted sequence.

### 4.3 Comparing correlations between samples

To test if the relationship between the GMLVQ-Scalar Projection and rate of future cognitive decline is significantly different between Development sample II and the Longitudinal prediction validation samples we used Fisher's r to Z transformation. To compare if the relationship of the GMLVQ-Scalar Projection and rate of future cognitive decline is significantly different between models using biological or cognitive data we generate a Steiger Z statistic (Steiger, 1980). See Supplementary *Methods Cross Validation Framework* for a compressive description of validation methodologies.

## 5 Results

### 5.1 Cognitive classification models for predicting sMCI vs. pMCI

**Fig. 2:**
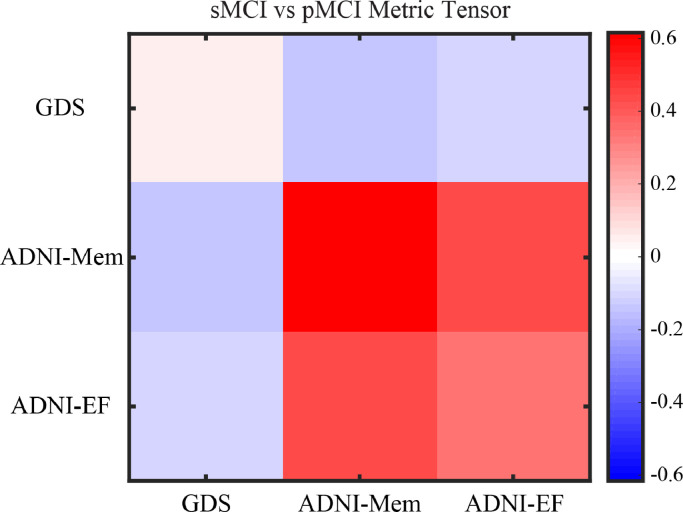
Cognitive classification Model - Metric Tensor Metric tensor for the classification model (sMCI vs pMCI) generated using cognitive data (GDS, ADNI-Mem, ADNI-EF). The colour scale indicates the predictive value for each cell in the metric tensor, where diagonal terms sum to 1. The diagonal terms show strong contribution of the ADNI-Mem score. The positive off diagonals terms indicate a positive interaction between the ADNI-Mem and ADNI-EF scores. The negative off diagonals terms indicate the negative interaction of the GDS score with the ADNI-Mem and ANDNI-EF scores. See also Figure S1 for examples of GMLVQ and possible interpretations (For interpretation of the references to colour in this figure legend, the reader is referred to the web version of this article.).

We tested whether a classification model that is based on the Generalised Metric Learning Vector Quantization (GMLVQ) framework and trained and tested on baseline cognitive data predicts progression from MCI to AD. In particular, we trained and tested both a linear and non-linear classifier to discriminate between sMCI and pMCI using cognitive data (Geriatric Depression Scale (GDS), ADNI Memory (ADNI-Mem) and ADNI Executive Function (ADNI-EF) from a sample of 167 MCI individuals (Development Sample II). We tuned the model with 2 hyper-parameters using nested cross validation and assessed its performance using 10-fold cross validation. The model successfully classified stable (sMCI; *n* = 113) vs. progressive (pMCI; *n* = 54) MCI individuals [Accuracy: 81.4%, MAE: 17.6%, TP: 84.9%, TN 79.8%]. We obtained identical performance by increasing model complexity to a non-linear classifier by increasing the number of prototypes per class to two [Accuracy:81.4%, MAE:17.6%, TP:84.9%, TN 79.8%], and therefore selected the linear model for further analysis. Interrogating the average metric tensor (Figure 2, Supplementary *Methods GMLVQ, Figure S1*) showed that the most predictive feature was ADNI-Mem (mean:0.55, std:+−0.12), while ADNI-EF (mean:0.35, std:+−0.09) and GDS (mean:0.1, std:+−0.05) had moderate and minor contributions to the classification task, respectively. These results suggest that the baseline ADNI-Mem score is the most discriminative cognitive feature for classifying sMCI vs. pMCI, as indicated by the diagonal terms in the metric tensor that are scaled to sum to one. Further, learning a metric in the input space of the classifier enables us to extend beyond the weighting of individual input features (such as ADNI-Mem score) and study the higher-order interplay between pairs of features with respect to the classification task. Interrogating the off diagonal terms of the metric tensor indicates that the interaction of GDS with ADNI-Mem or ADNI-EF is important for classifying sMCI vs. pMCI individuals. The positive off-diagonal terms indicate a positive interaction between the ADNI-Mem and ADNI-EF scores that group individuals from the same class. In contrast, the negative off diagonal terms indicate that the GDS score has a negative interaction with the ADNI-Mem and ADNI-EF scores and separate individuals into different classes. For example, individuals with similar baseline ADNI-Mem and ADNI-EF scores may be classified in different groups depending on their baseline GDS score, with higher scores likely reflecting affective disturbance and MCI comorbidity.

### 5.2 Composite grey matter score for predicting cross-modality associations

**Fig. 3:**
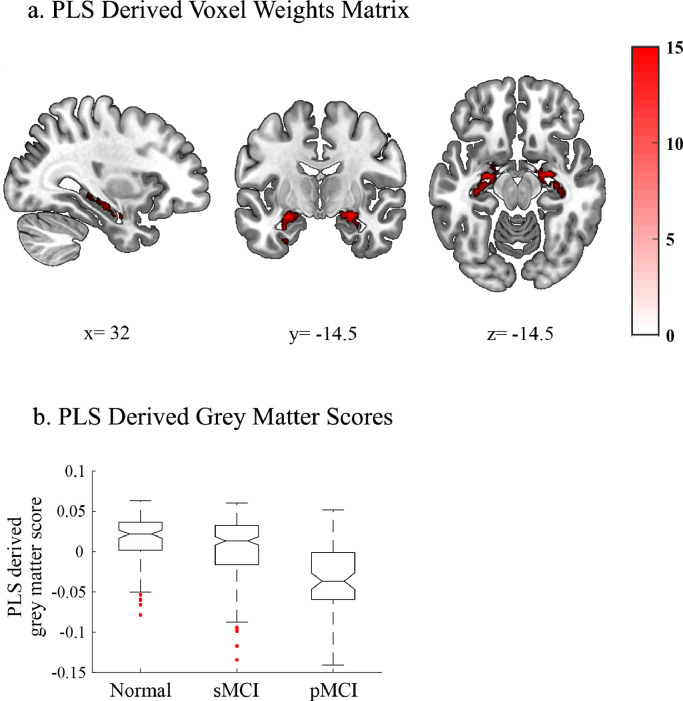
PLS modelling of ADNI-Mem **a. PLS derived voxel weights matrix**. Voxel weights are derived using the PLSr-RFE methodology. Retained voxels are overlaid on the MNI template in neurological convention (left is left). The colour scale represents the average z-statistic of weights per voxel across all cross folds. All retained voxels are red indicating positive weights. **Table S2** lists the anatomical regions and voxel weights for the PLS voxel matrix. The x, y and z coordinates denote the location of the sagittal, coronal and axial slices, respectively. **b. PLS derived grey matter scores for cognitively normal, sMCI and pMCI groups:** Boxplots of the PLS derived grey matter scores for cognitively normal, sMCI and pMCI groups. The centre line represents the median and the edges of the boxes represent the 25th and 75th percentiles of each sample. The medians of two samples are significantly different at the *p*<0.05 if the edge of the intervals around each notch do not overlap. Red points denote outliers (For interpretation of the references to colour in this figure legend, the reader is referred to the web version of this article.).

We next determined the spatial distribution and weight of grey matter voxels that are associated with memory loss in AD. We used PLSr-RFE on data from cognitively normal and MCI individuals (Development Sample), to derive latent features based on whole-brain grey matter that predict baseline ADNI-Mem, as this was shown to be the most discriminative cognitive feature for classifying sMCI vs. pMCI individuals. We determined the optimal number of grey matter voxels and PLS dimensions to retain, using nested cross validation within each of 5 cross folds. We observed that the predictive voxels aggregated within the medial temporal cortex (Figure 3a, Supplementary *Table S3*) and that a single PLS dimension explained comparable variance in the ADNI Mem score in both training [r^2^(587) = 0.1855, *P* < 0.0001] and test [r^2^(587) = 0.1756, *P* < 0.0001] sets (Supplementary *Figure S4a*, Supplementary *Table S2a*). No other PLS components were retained following cross validation.

Next, we derived a PLS derived grey matter score for a validation sample that did not include individuals that were used in the model development (Cross-modal associations validation sample I). This value represents the weighted linear sum of grey matter voxels that best described the ADNI-Mem score in the Development sample. We showed that this score accounts significantly for variance in ADNI-Mem for the Cross-modal associations validation sample I (*n* = 219) that was not previously used in the PLSr-RFE feature generation ([*r*^2^(217) = 0.33, *P* < 0.0001]). This relationship remained significant when we controlled for (Age; r^2^(216) = 16%, *P*<0.0001, Gender; r^2^(216) = 24% *P*<0.0001, or Education; r^2^(216) =37% *P*<0.0001). It is likely that the higher variance explained by the PLS derived grey matter score for the validation sample I relative to the development sample is due to the significantly higher degree of atrophy (lower PLS grey matter score) in the validation sample I (Wilcoxon Signed Rank; *z*=−3.42, *p*<0.0001). That is, a greater amount of variance in ADNI-Mem is likely explained by the greater amount of AD related atrophy in the validation sample. Further, we observed significant differences (independent sample t-tests) in the PLS derived grey matter score between three sub-groups (cognitively normal, sMCI and pMCI) within the Development sample used in the PLSr-RFE analysis. In particular, the cognitively normal group showed significantly higher scores than the pMCI group (t(170)=9.13, *P*<0.0001, Cohens *D* = 1.5) and the sMCI showed significantly higher score than pMCI group (t(165)=5.7, *P*<0.0001, Cohens *D* = 0.94). However, when comparing cognitively normal vs. sMCI individuals we observed only a small effect [t(230)=3.7, *P* = 0.00072 (FWE Corrected), Cohens *D* = 0.48] (Figure 3b). Taken together these results suggest that the PLS derived grey matter score captures variance that relates to memory dysfunction (i.e. poor ADNI-Mem scores) due to AD.

We next compared the variance explained in ADNI-Mem by the PLS derived grey matter score to the variance explained by the average grey matter density in medial temporal regions (i.e. amygdala, hippocampus) known to be related to ADNI-Mem (Nho et al., 2012). For each test set within the nested cross-validation framework, we extracted mean grey matter density from regions in the amygdala and hippocampus, as defined using the Brainnetome atlas (Fan et al., 2016). We then compared the variance explained in ADNI-Mem for these a-priori selected regions with the variance explained by the PLS derived grey matter feature. We observed that the PLS derived grey matter score explained significantly more variance in the ADNI-Mem score than the mean grey matter density from a-priori selected regions in the medial temporal cortex (t(24)=5.6, Cohens *D* = 1.12, *P*<0.0001) (Supplementary *Table S4*). This finding suggests that the multivariate relationship between grey matter voxels captured by the PLS accounts for higher variability in individual ADNI-Mem scores than the average grey matter density in brain regions defined by coarser parcellations.

Finally, we tested whether the PLS derived grey matter score differs across individuals that vary in cortical tau pathology, as measured by FTP-PET (Table 1). Comparing individuals from an independent sample (Cross-modal associations validation sample) with tau positive vs. tau negative scores (independent samples *t*-test) showed the strongest effect within Braak stage 12 [t(444)=9.6, *P*<0.0001 Cohens *D* = 1.9]. Further, the PLS derived grey matter score correlated (Pearson's correlation) significantly with cortical tau burden across all individuals, with the strongest effect for Braak stage 12 [r^2^(444) = 0.32, *P*< 0.0001]. These results suggest that the PLS derived grey matter score relates to both memory deficits and tau deposition associated with AD. These results are consistent with previous studies showing a strong relationship between memory, medial temporal lobe atrophy, and regional (or Braak 12 stage) deposition of tau (Cho et al., 2016; Harrison et al., 2019; Johnson et al., 2016; Knopman et al., 2019; Schöll et al., 2016).

**Table 1:** (PLS derived grey matter score relationship with flortaucipir PET Tau) relationship of the PLS grey matter score with flortaucipir Tau measures. The table shows the threshold for tau positivity for each of the Braak stages, the statistical differences between the grey matter scores for tau positive vs. tau negative individuals, and the correlation of the PLS grey matter score with flortaucipir tau across all individuals.

Braak stage	Threshold	Tau positive vs Tau negative				GM score vs Tau
		p	t	Cohen d	Pos/Neg	r^2^
**tau Braak 12**	1.95	<0.0001	9.7	1.9	27/419	0.32
**tau Braak 34**	1.89	<0.0001	8.3	1.5	33/413	0.15
**tau Braak 56**	1.93	<0.0001	5.7	1.3	21/425	0.08

### 5.3 Comparing the performance of biological vs. cognitive models

**Fig. 4:**
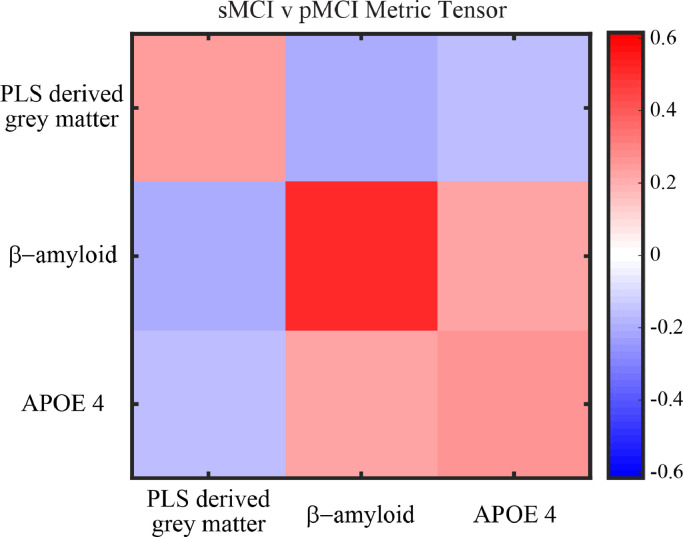
Biological Classification Model - Metric Tensor Metric tensor for the classification model (sMCI vs pMCI) generated using biological data (PLS derived grey matter score, β-amyloid, APOE 4). The colour scale indicates the predictive value for each cell in the metric tensor, where diagonal terms sum to 1. The diagonal terms show strong contribution of β-amyloid. The positive off diagonals terms indicate a positive interaction between β-amyloid and APOE 4. The negative off diagonals terms indicate the negative interaction of the PLS derived grey matter score with both β-amyloid and APOE 4. See also Figure S1 for examples of GMLVQ and possible interpretations (For interpretation of the references to colour in this figure legend, the reader is referred to the web version of this article.).

We tested whether a classification model trained and tested on baseline biological data discriminates sMCI vs. pMCI. We developed a biological classification model of similar complexity to the cognitive model (i.e. linear classifier (1 prototype per class), 3 features, 2 hyper parameters) based on the same data sample (Development Sample II, *n* = 167) using as predictors: PLS derived grey matter score, β-amyloid burden (measured by FBP-PET) and APOE 4 status (positive: presence of 1 or 2 APOE4 alleles, negative: no APOE4 alleles). The model successfully discriminated between sMCI vs. pMCI individuals [Accuracy: 81.9%, MAE: 18.3%, True Positive: 81.1%, True Negative 82.3%]. We observed comparable classification performance when we increased the complexity of the biological model to a non-linear classifier (2 prototypes per class) [Accuracy: 80.7%, MAE: 19.2%, True Positive: 81.1%, True Negative: 80.5%]. The metric tensor of the model (Figure 4) indicates that the feature with the highest predictive value is baseline β-amyloid burden (mean:0.48, std:+−0.16), with similar contributions from baseline PLS derived grey matter (mean:0.28, std: +- 0.14) and APOE4 status (mean:0.24, std: +−0.10). Further, interrogating the off diagonal terms of the metric tensor indicated a positive interaction between baseline β-amyloid burden and APOE 4 status; that is baseline β-amyloid burden and APOE 4 status groups individuals from the same class. In contrast, we observed a negative interaction between baseline β-amyloid burden and the baseline PLS derived grey matter score; that is, the combination of these features separates sMCI from pMCI individuals. For example, individuals with high baseline β-amyloid burden and low baseline PLS derived grey matter score (i.e. low grey matter density in medial temporal areas) are grouped in separate classes (sMCI vs. pMCI) from individuals with high baseline PLS derived grey matter score (i.e. high grey matter density) and low baseline β-amyloid burden. Finally, we observed no significant differences (t-tests across cross folds) in classification performance between the cognitive and biological models (Accuracy: [t(9)=−0.13, *P* = 0.90], MAE: [t(9)=0.17, *P* = 0.87], True Positive: [t(9)=0.54, *P* = 0.60], True Negative: [t(9)=−0.32, *P* = 0.75]), suggesting that baseline cognitive and biological features contribute similarly to the binary classification of sMCI vs. pMCI individuals.

### 5.4 Trajectory modelling: predicting individual variability in the rate of future cognitive decline

**Fig. 5:**
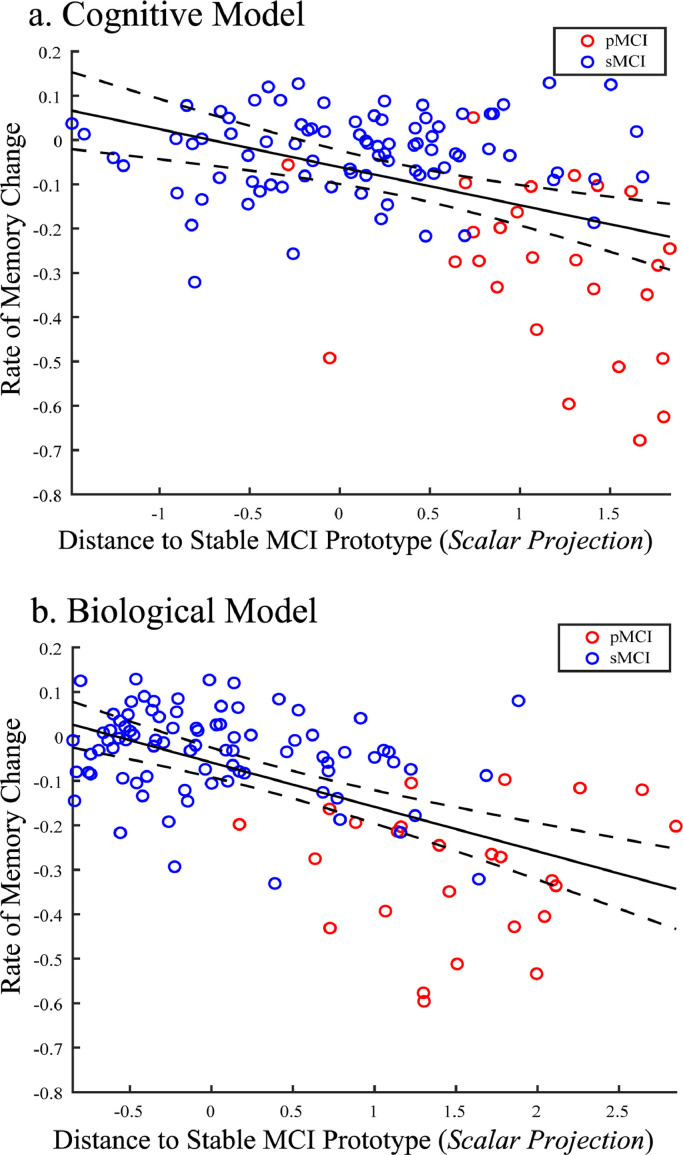
Correlating GMLVQ-Scalar projections with rate of memory change: Correlation of the GMLVQ-scalar projections derived from the a) cognitive model, b) biological model with the rate of ADNI-Mem change for Development Sample I. Red dots indicate pMCI individuals, blue dots indicate sMCI individuals. The central black line is the regression line for the fit of the GMLVQ-scalar projection to the rate of ADNI-Mem change; the dashed lines represent the 95% confidence intervals for this regression line. Data used to train the model (*n* = 52) were not used to test the relationship between the scalar projection and rates of future cognitive decline and are not shown here.

Our analyses so far have focused on binary classifications (i.e. sMCI vs. pMCI). However, this approach is limited, as it assumes distinct patient classes and does not capture dynamic changes in disease progression over time. To extend beyond this binary framework, we developed a trajectory modelling approach by deriving a continuous metric based on a GMLVQ-scalar projection (i.e. distance of each MCI patient from the sMCI prototype) and using only baseline data. We then confirmed that this projection relates to individual variability in the rate of future cognitive decline. In particular, we defined the rate of future cognitive decline as the rate of change in the ADNI-Mem scores across measurements following baseline, where baseline is defined as the date of the FBP-PET scan used as a predictor for deriving the GMLVQ-scalar projection. We focussed on change in memory performance as measured by ADNI-Mem, as a) memory decline has been shown to occur prior to decline in other cognitive domains in sporadic AD, b) our metric leaning model showed that ADNI-Mem was the most discriminative cognitive feature for the sMCI vs. pMCI classification compared to the other cognitive variables tested (GDS, ADNI-EF). We then tested whether this prognostic metric of future cognitive decline differs for cognitive vs. biological models. For the same sample used in the binary classifications (Development Sample II) we observed that scalar projections derived from either the cognitive or the biological model account significantly for variance in the rate of future memory decline (Figure 5) (i.e. Cognitive: [r(165) = −0.41 (95% CI: [−0.51 −0.32]), *P* < 0.0001], Biological: [r(165) = −0.55 (95% CI: [−0.62 −0.47]), *P* < 0.0001]). Further analyses showed that our trajectory modelling approach can be extended to predict data with less than 3 years of clinical diagnosis (Supplementary *Figure S5*) and future rate of cognitive decline as measured by standard clinical scales (i.e. MMSE; Supplementary *Figure S6*), providing evidence for the clinical relevance of our approach.

**Fig. 6:**
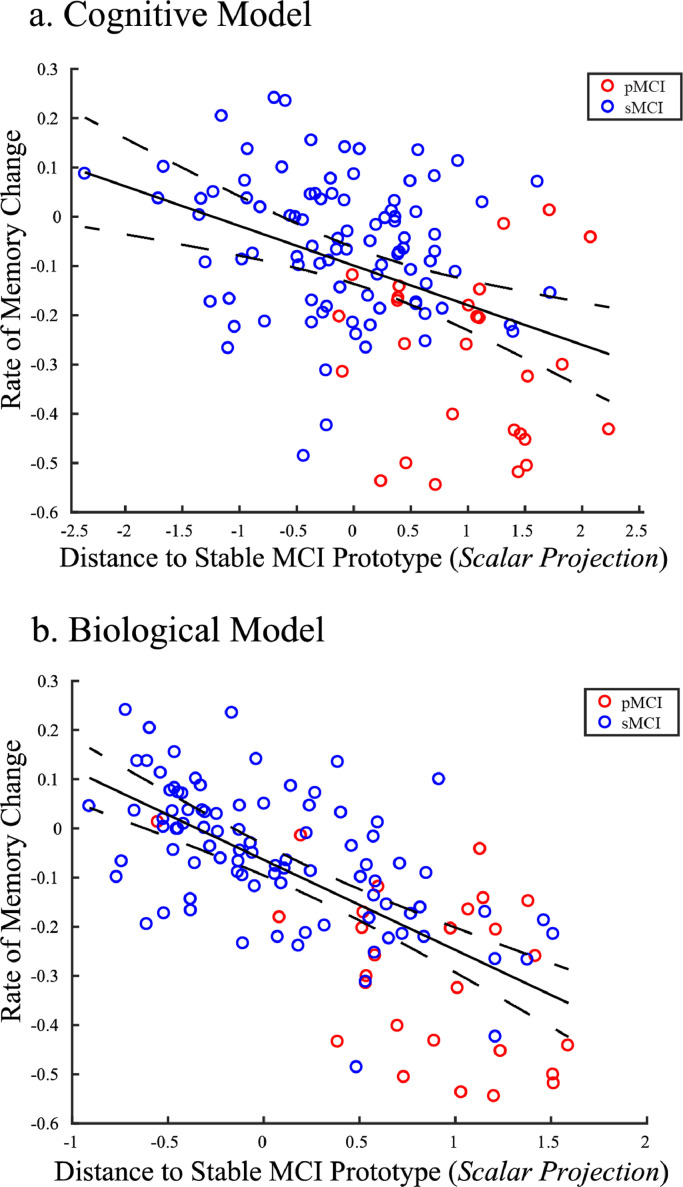
Correlating Scalar projections from Cognitive and Biological Models with rate of memory change: Out-of-sample Validation Correlation of scalar projection derived from the a) cognitive model, b) biological model with the rate of ADNI-Mem change for the longitudinal validation data set. Red dots indicate pMCI individuals, blue dots sMCI individuals. The central black line is the regression line for the fit of the GMLVQ-scalar projection to the rate of ADNI-Mem change; the dashed lines represent the 95% confidence intervals for this regression line. Outliers identified by the Robust Correlation toolbox (cognitive *n* = 7, Biological *n* = 8) are not shown for illustrative purposes. Note that this validation sample includes data from 3 β-amyloid negative pMCI individuals who had a scalar projection of less than 0.25 (i.e. very close to the sMCI prototype). Investigating the relationship of the scalar projection to future cognitive decline for these individuals showed dissociable cognitive trajectories from most pMCI individuals.

Further, we validated the relationship of the GMLVQ-scalar projection with cognitive decline for individuals with MCI against a new independent validation data sample (Longitudinal prediction validation sample). To calculate the scalar projections, we chose the metric tensor and prototype positions from the cognitive or biological models with the median test performance across resampling using the Development Sample II. To generate a baseline PLS derived grey matter score for the Longitudinal prediction validation sample, we multiplied the voxel weights matrix determined by PLSr-RFE on the Development sample (Figure 3a) with grey matter density from baseline structural scans for the longitudinal prediction validation sample (i.e. data not used for the PLSr-RFE feature generation). We observed a significant correlation of the scalar projection with the rate of future ADNI-Mem change for cognitive data [r(124) = −0.4, (95% CI: [−0.55 −0.25]), *P* < 0.0001]. This relationship remained significant when we controlled for: Age; [r(123) = −0.32, (95% CI: [−0.47 −0.14]), *P* = 0.0003], Gender; [r(123) = −0.4, (95% CI: [−0.55 −0.22]), *P*< 0.0001], or Education; [r(123) = −0.4, (95% CI: [−0.54 −0.23]), *P*< 0.0001]). Further, we observed a significant correlation of the scalar projection with the rate of future ADNI-Mem change for biological data [r(124) = −0.68, (95% CI: [−0.76 −0.58]), *P* < 0.0001] (Figure 6). This relationship remained significant when we controlled for: Age; [r(123) = −0.57, (95% CI: [−0.67 −0.46]), *P*< 0.0001], Gender; [r(123) = −0.63, (95% CI: [−0.71 −0.53]), *P*< 0.0001], Education; [r(123) = −0.63, (95% CI: [−0.73 −0.53]), *P*< 0.0001]. This relationship was not significantly different between Development Sample I vs. Longitudinal prediction validation samples (Fisher's r to Z, Cognitive model: [*Z*=−0.1, *P* = 0.92], Biological model: [*Z*=−1.76, *P* = 0.08]). Further, correlations between the scalar projection and the rate of future memory decline were significantly stronger for biological compared to cognitive models (Steiger's Z, [*Z*=−3.86, *P*<0.0001]). This difference between models remained significant when we controlled for Age; (Steiger's Z, [*Z*=−3.33, *P* = 0.0004]), Gender; (Steiger's Z, [*Z*=−3.57, *P* = 0.0002]), or Education; (Steiger's Z, [*Z*=−3.57, *P* = 0.0002]). Taken together these findings suggest that the biological model explains significantly larger variance in the rate of future memory decline than the cognitive model.

Finally, we tested whether our trajectory modelling approach delivers stronger predictions when including non-invasively measured biological data to the basic baseline cognitive model. Adding the baseline PLS derived grey matter feature and APOE 4 status to the cognitive model showed a substantial increase in the variance in the rate of future memory change explained by the scalar projection (Table 2). These results suggest that predicting the rate of future cognitive decline is enhanced by adding non-invasively measured baseline biological features to baseline cognitive data.

**Table 2:** Correlations of scalar projections with the rate of ADNI-Mem change for models based on cognitive and / or biological data. Pearson's correlation coefficients are shown for Development Sample (b) based on cross-validation and the independent data used for out of sample validation (longitudinal validation sample).

Data type	Pearson's r [95% C.I] Cross validation	Pearson's r out-of-sample validation
Biological: (GM+APOE4+ β-Amyloid)	−0.55 [−0.66 −0.53]	−0.68 [−0.76 −0.58]
Cognitive: (GDS+ADNI-Mem+ADNI-EF)	−0.41 [−0.5 −0.30]	−0.4 [−0.55 −0.25]
Cognitive+GM	−0.46 [−0.52 −0.34]	−0.49 [−0.61 −0.35]
Cognitive+APOE 4	−0.47 [−0.55 −0.38]	−0.48 [−0.61 −0.33]
Cognitive+GM+APOE 4	−0.5 [−0.57 −0.42]	−0.53 [−0.64 −0.4]

## 6 Discussion

Despite the importance of early diagnosis of Alzheimer's disease for clinical practice and treatment, we still lack robust tools for predicting individual progression to dementia. The multimodal longitudinal measurements across large-scale samples available in ADNI provide a testbed for machine learning approaches that generate predictive features and discriminate between patient groups (Weiner et al., 2017, 2015). Here, we propose a novel trajectory modelling approach based on an integrated feature generation and classification methodology that predicts individual disease trajectories based on continuous measures of cognitive decline. Our modelling approach is in line with the current 2018 NIA-AA research framework that defines AD as a continuum and advances the state-of-the-art and clinical validity of machine learning applications to the prediction of dementia due to AD in the following main respects.

First, we successfully predict whether individuals will progress from MCI to dementia due to AD, employing a transparent machine learning approach (i.e. prototype based classifier with metric learning and linear decision boundary) trained on informative and interpretable baseline cognitive data. We show that baseline composite scores related to memory and executive function (ADNI-Mem, ADNI-EF composite score) are highly predictive of disease progression. The high cross-validated classification performance of our model is in line with previous studies showing that similar neuropsychological data are predictive of MCI progression to dementia due to AD (Belleville et al., 2017; Chapman et al., 2011; Pereira et al., 2018; 2017; Silva et al., 2013; Tabert et al., 2006). Further, we demonstrate a negative interaction between baseline cognitive (memory, executive function) and affective scores that separates individuals into different classes, with higher baseline affective scores potentially reflecting MCI comorbidity. Previous studies have shown that moderate to severe depressive symptoms (i.e. GDS> 15) are predictive of MCI conversion to AD (Defrancesco et al., 2017), while mild depressive symptoms do not increase conversion risk (Chen et al., 2008; Defrancesco et al., 2017). Here, we show that the interaction between scores that are indicative of mild depression (i.e. GDS<10) and memory dysfunction discriminates stable from progressive MCI individuals. Thus, our metric learning approach on multimodal data (i.e. cognitive and affective measurements) may provide a means of reducing MCI patient misclassification due to comorbidity (e.g. affective disturbance).

Second, we developed a supervised feature generation method (PLSr-RFE) that allows us to derive predictive and interpretable biomarkers based on structural brain imaging data. We demonstrate that grey matter density in the medial temporal lobe predicts variability in memory scores (i.e. ADNI-Mem score). In particular, this grey matter score is shown to be a highly predictive feature for the classification of sMCI vs. pMCI individuals, consistent with previous studies showing that grey matter density in the medial temporal lobe (MTL) is associated with AD (Davatzikos et al., 2009; Hedden and Gabrieli, 2004; Mak et al., 2017; Matsuda, 2016; Rathore et al., 2017) and ADNI-Mem scores (Nho et al., 2012). Previous work using a similar PLS methodology (sparse PLS) showed a similar spatial pattern of grey matter voxels that are predictive of MMSE scores (Monteiro et al., 2016). Extending beyond this work, we generate a biomarker based on a projection (PLS-derived grey matter score) that is shown to explain more variance in ADNI-Mem scores than the grey matter density estimated from the corresponding atlas-defined MTL region. Importantly, we show that this PLS-derived biomarker predicts cortical tau pathology as measured by PET, providing a strong link between regional brain atrophy, memory decline, and tau pathology (Maass et al., 2018). Thus, our PLSr-RFE methodology has the potential to enhance interoperability across cohorts that typically include grey matter measurements (i.e. structural MRI scans) but may vary in the inclusion of other variables (e.g. cognitive or tau measurements). Here, we focused on grey matter density (un-modulated data), as it has been suggested to reflect mesoscopic grey matter thinning (Radua et al., 2014) that is evident in AD (Jagust, 2018). The same PLSr-RFE methodology can be extended to a wider range of measures derived from structural MRI scans (e.g. variation in cortical volume, shape and texture) that have been shown to be predictive of AD (for reviews: (Leandrou et al., 2018; Mateos-Pérez et al., 2018; Matsuda, 2016)).

Third, our trajectory modelling approach (GMLVQ-Scalar Projection) extends beyond binary patient classification approaches (Rathore et al., 2017) that are poorly constrained. Recent methodological frameworks for mining neuroimaging data (Jollans et al., 2019) and predicting progression to AD (Samper-González et al., 2018) have focused on binary classifications that are based on discrete clinical labels (i.e. stable vs. progressive MCI), as determined by arbitrary criteria (e.g. within a 3 year period of clinical assessment). As a result, these approaches are limited by risk of patient misclassification. That is, patients at the class boundary with different disease trajectories may be classed in the same MCI group (e.g. a patient who progresses to AD within 1 day from clinical assessment and a patient who converts in 3 years will be classified as pMCI). Similarly, patients with similar disease trajectories may be classified in different MCI groups (e.g. a patient who converts in 3 years will be classified as pMCI, while a patient who remains stable for 3 years and progresses to dementia 1 day after the clinical assessment will be classified as sMCI). To overcome this limitation and make meaningful predictions in AD, modelling approaches need to capture continuous information in prognostic trajectories and consider target uncertainty (i.e. the future clinical diagnosis) (for review (Janssen et al., 2018)). Although recent time-to-event models (e.g. survival analysis models predicting time to conversion) (Alsaedi et al., 2018; Casanova et al., 2013; Desikan et al., 2010; Jack et al., 2010; Landau et al., 2010; Liu et al., 2017; Michaud et al., 2017; Oulhaj et al., 2009; Young et al., 2014) capture continuous information in patient trajectories they are limited by target uncertainty; that is, estimating the exact time to conversion is limited by the frequency of clinical follow-ups and poor inter-rater reliability (i.e. diagnoses may differ across clinicians).

Our trajectory modelling approach predicts future ADNI-Mem scores based on baseline data, allowing us to capture individual disease trajectories and reducing the risk of patient misclassification. In particular, we derive continuous prognostic scores of individual cognitive decline (i.e. scalar projection) by training the model based on ‘noisy’ diagnostic labels (i.e. patient classes that are poorly defined e.g. sMCI vs pMCI). As our metric learning model has limited freedom (linear low-parameter model), separating continuous target values (i.e. individualised cognitive trajectories) into two broad classes (sMCI vs. pMCI) forces the model to extract key underlying structures in the data that distinguish between target values, ignoring subtle differences in target values. Further, employing separate feature generation (i.e. PLSr-RFE) and classification (GMLVQ scalar projection) stages allows us to interrogate interpretable predictive features of progression to AD and derive predictions that generalise to patient data from independent samples from the model development sample. This is in contrast to deep learning methods that require large training samples and are shown to be difficult to interpret and generalise (Davatzikos, 2019), raising questions about the clinical utility of these approaches (for review (Topol, 2019)).

Comparing our trajectory modelling methodology to binary classifications on the same data (i.e. cognitive vs. biological) shows dissociable results. A binary metric learning algorithm shows similar performance in the binary classification of MCI subgroups (sMCI vs. pMCI) when trained on baseline cognitive vs. biological data. In contrast, the scalar projection derived from biological data explains significantly higher individual variability in the rate of future cognitive decline than the scalar projection derived from cognitive data. Further, we demonstrate that the predictive power of our trajectory modelling methodology is enhanced when including non-invasively measured baseline biological data in addition to baseline cognitive data. Although our model shows high accuracy of cognitive decline when trained on cognitive data, there is a substantial gain in predictive efficacy when adding baseline data on APOE 4 status or grey matter density (PLS derived grey mater scores). This is consistent with previous studies (Dukart et al., 2015; Thung et al., 2018; Vogel et al., 2018) showing enhanced prediction of time to AD conversion when including biological compared to neuropsychological data alone.

Previous work on trajectory modelling has focused on discretising continuous values (i.e. future change in cognitive scores) into latent classes that are then used as outcome measures in classification models (Bhagwat et al., 2018; Hochstetler et al., 2015; Wang et al., 2019; Wilkosz et al., 2010). For example, previous studies (Bhagwat et al., 2018) used machine learning (i.e. longitudinal Siamese neural-network) to fuse baseline and follow up imaging and clinical scores to predict whether individuals will decline fast or slow (based on MMSE scores) or fast, moderate or slow (based on ADAS-cog). Further studies (Hochstetler et al., 2015) used classification and regression trees on baseline demographic, lifestyle, cognitive and biological data to classify individuals in three latent classes (fast, medium or slow decline) with similar growth patterns of cognitive and functional changes, while others (Wilkosz et al., 2010) used latent class trajectory models to derive six different trajectories for cognitive and behavioural decline due to AD. However, the generalisability and interoperability of these approaches have been recently questioned (Wang et al., 2019). Our trajectory modelling approach differs from this previous work, as it avoids assumptions related to discretising continuous values. In particular, we derive a continuous metric (i.e. scalar projection) from a discrete classification model (i.e. metric learning) that predicts individual rates of future cognitive change (i.e. change in ADNI-Mem). Finally, our approach is in line with previous work predicting exact changes in MMSE or ADAS-Cog scores (Fan et al., 2008; Zhang et al., 2012). In particular, previous studies used baseline and follow-up structural MRI and FDG-PET data to predict future scores on cognitive tests at different time intervals (Zhang et al., 2012), or structural MRI to predict the rate of change in MMSE scores (Fan et al., 2008). Our modelling approach differs from this previous work in fusing baseline multimodal data into a single metric (i.e. scalar projection) to predict future rates of cognitive change (i.e. change in ADNI-Mem, or MMSE scores).

In sum, we propose a robust methodology based on modelling multimodal data that determines predictive and interpretable markers of individual variability in progression to dementia due to AD. Although our investigations have focused on amnestic MCI, our methodology has the potential to be extended to predict individual disease trajectories specific to AD subtypes, following recent work modelling neuroimaging data (Dong et al., 2016; Young et al., 2018). Further, previous work on preclinical populations has investigated the role of grey matter atrophy and cortical amyloid burden in future cognitive decline (Bilgel et al., 2018; Burnham et al., 2016; Dumurgier et al., 2017; Insel et al., 2015). Extending our trajectory modelling approach to preclinical populations using multimodal data has high clinical relevance, especially as clinical trials are moving towards less severely affected individuals who are unlikely to progress over the short time scales of clinical trials (Bilgel et al., 2017, 2014; Grober et al., 2008; Mormino et al., 2014). Thus, our approach has strong potential to deliver tools of high clinical relevance that reduce patient misclassification and facilitate effective stratification of individuals to prognostic or treatment pathways and clinical trials based on individualised rates of cognitive decline.

## Funding

This work was supported by grants to Z.K. from the Biotechnology and Biological Sciences Research Council (H012508 and BB/P021255/1), Alan Turing Institute (TU/B/000, 095), Wellcome Trust (205,067/Z/16/Z) and to Z.K. and W.J. from the Global Alliance.

## CRediT authorship contribution statement

**Joseph Giorgio:** Conceptualization, Formal analysis, Investigation, Methodology, Writing - original draft, Writing - review & editing. **Susan M. Landau:** Conceptualization, Data curation, Formal analysis, Investigation, Writing - original draft, Writing - review & editing. **William J. Jagust:** Conceptualization, Data curation, Investigation, Writing - original draft, Writing - review & editing. **Peter Tino:** Conceptualization, Investigation, Methodology, Writing - original draft, Writing - review & editing. **Zoe Kourtzi:** Conceptualization, Investigation, Methodology, Writing - original draft, Writing - review & editing.

## Declaration of Competing Interest

The authors declare no competing interests.

## Appendix Supplementary materials

Image, application 1
