# Supplementary material for: Modelling prognostic trajectories of cognitive decline due to Alzheimer's disease
Source: Neuroimage Clin. 2020 Jan 26;26:102199. doi: 10.1016/j.nicl.2020.102199 (PMC7044529; doi:10.1016/j.nicl.2020.102199)
Supplement: Supplementary file 1 [file mmc1.docx]

**Supplementary Material**

**Modelling prognostic trajectories of cognitive decline due to Alzheimer’s disease**

Joseph Giorgio^1^, Susan Landau^2^, William Jagust^2^, Peter Tino^3^, Zoe Kourtzi^1^, for the Alzheimer’s Disease Neuroimaging Initiative

**Supplementary Methods**

**ADNI Participants**

**Table S1. Sample Demographics**

| Development Sample:  589 individuals (baseline diagnoses: Normal =317, MCI=272) | |
| --- | --- |
| **Age (mean+-std)** | 73.3+-7.1 |
| **Education (mean+-std)** | 16.3+-2.6 |
| **Gender (Male/Female)** | 289/300 |
| **APOE 4 Status (neg/pos)** | 376/207* |
| Longitudinal prediction validation sample:  126 individuals (baseline diagnoses: MCI=126) | |
| **Age (mean+-std)** | 71.6+-7 |
| **Education (mean+-std)** | 16.3+-2.7 |
| **Gender (Male/Female)** | 74/52 |
| **APOE 4 Status (neg/pos)** | 61/65 |
| Cross-modal associations validation sample  446 individuals (Normal=263, MCI=172, AD=11) | |
| **Age (mean+-std)** | 74.7+-7.4 |
| **Education (mean+-std)** | 16.5+-2.5 |
| **Gender (Male/Female)** | 213/233 |
| **APOE 4 Status (neg/pos)** | NA |

*Table S1: demographic information at baseline measurement including age, education in years, Gender and APOE 4 status for the 3 data samples used in our study*

**6 Individuals missing APOE 4 data*

***Methods PLS***

***Partial Least Squares regression (PLSr)***

To determine the grey matter voxels that predict ADNI-Mem we used PLS regression. PLS regression is a technique that determines multivariate relationships between predictor variables to best describe response variables. PLS regression applies a decomposition on a set of predictors to create latent variables that show the maximum covariance with the response variables (Krishnan et al., 2011; McIntosh and Lobaugh, 2004).

For each iteration we ran the PLS regression to derive 1 to 5 latent variables (new co-ordinates axis or “dimensions”) within the predictor feature space. To assess the stability of the predictor loadings (i.e. weights obtained as projections of input vectors onto the latent variables) we generated 200 bootstrap samples from our data. We performed a PLS regression for each bootstrap sample to generate a distribution of weights per voxel. To generate these distributions, we first corrected the estimated components for axis rotation and reflection across bootstrap samples using Procrustes rotation (Milan and Whittaker, 1995).

While PLS is robust so that the number of predictors can be more than the number of observations, the extremely high variance explained by the complete model can show that merely having many predictors can explain “erroneous” variance in the outcome variable. Standard methods to test for model acceptance involve permutation testing to generate a null hypothesis (McIntosh and Lobaugh, 2004). Here, we accepted a model, specifically a given PLS dimension, based on its out-of-sample generalisation. We assessed this by learning a scale invariant voxel weights matrix (variance normalised loadings matrix) on training data and generating a given PLS score for a test set by multiplying the voxel weights matrix by the test data. Finally, we tested the variance that can be explained by this PLS score in our predictor variable.

***PLSr Recursive Feature Elimination (PLSr-RFE)***

We performed recursive feature elimination, whereby we iteratively removed voxels (original input features) that have weak predictive value. While recursive feature elimination is generally used for feature selection, we performed a simultaneous feature selection and feature construction method using the PLSr recursive elimination framework. To eliminate features we calculated a variance-normalised weight for each voxel ${Zw}_{vox,dim}$ akin to a z-statistic.

${Zw}_{vox,dim}= \frac{\mu(bootstrap)}{\sigma(bootstrap)}$ where the voxel location is indexed by $vox\in\left\{ 1,\ldots,V \right\}$ , the PLS dimension is indexed by$dim\in\left\{ 1,\ldots,D \right\}$ and $bootstrap$ is the distribution of weights for the given voxel and dimension across bootstrapped samples. i.e. The mean weight across all bootstrap samples was normalised by the corresponding standard deviation.

As the weights can be either negative or positive, we calculated the stability ${Sw}_{vox}$ of a voxel by the sum of the absolute z values across the PLS dimensions.

$${Sw}_{vox}=\sum_{dim=1}^{D} \left| {Zw}_{vox,dim} \right|$$

In order to determine the least stable voxels to be eliminated in each iteration, we ranked each voxel by its stability from lowest to highest and removed the lowest 25^th^ percentile.

We then repeated this process iteratively removing the least stable 25^th^ percentile of voxels. After each reduction we tested how well the model using the remaining voxels predicts the response variable. This value was generated using a nested cross validation framework. To determine the optimal number of voxels, we implemented an early stopping paradigm, where we tested after each iteration of the recursive feature elimination loop the generalisation performance of the learnt PLS dimensions generated from the voxel subset. We simultaneously assessed the performance of each PLS dimension to generalise out-of-sample and the “optimal” number of voxels required to generate these dimensions.

We showed both within-sample (Development Sample) generalisation (i.e. test performance across the 5-fold cross-validation test set) and out-of-sample generalisation (Cross-modal associations validation sample). We tested the cross modal associations of the PLS derived grey matter score with ADNI-Mem scores using the Cross-modal associations validation sample I, as these individuals were not used for model formulation. Further, we tested the cross modal associations of the PLS derived grey matter score with cortical tau burden (measured by FTP-PET) using the Cross-modal associations validation sample.

***Methods GMLVQ***

***LVQ***

Learning Vector Quantisation (LVQ) comprises classifiers that operate in a supervised manner to iteratively modify class-specific prototypes to find boundaries of discrete classes. LVQ classifiers are defined by a set of vectors (prototypes) that represent classes within the input space. These prototypes were updated iteratively throughout the training phase, resulting in changes in class boundaries. For each training example the closest prototype for each class was determined. These prototypes are then updated so that the closest prototype representing the same class as the input example is moved towards the training example and those representing different classes are moved further away.

Training data sample of size *n* is denoted by $\left( x_{i},y_{i} \right) \in\mathbb{R}^{m}\boldsymbol{\times}\left\{ 1\boldsymbol{,\ldots,}K \right\}\mathbf{,}i=1,2,\ldots,n$, where *m* denotes data dimensionality and *K* the number of different classes. In this instance the LVQ network comprises *L* prototypes $w_{q}\in\mathbb{R}^{m}, q=1,2,\ldots,L$defined by their location in the input space and their class label $c\left( w_{q} \right) \in\left\{ 1,\ldots,K \right\}.$

Prototypes are adapted automatically throughout training so that distances between points of class $c\in\left\{ 1,\ldots,K \right\}$ and their corresponding prototypes sharing the same label *c* is minimised, while keeping the prototypes of other classes as far as possible. Classification is then performed on test data based on a winner-takes-all process, whereby a previously unseen input vector $x_{i} \in\mathbb{R}^{m}$ is assigned the class label $c\left( w_{q} \right)$ of the closest prototype $\left( w_{q} \right)$.

***GMLVQ***

The Generalised Matrix LVQ (GMLVQ) (Schneider et al., 2009) extends the LVQ utilising a full metric-tensor for a more robust (with respect to the classification task) distance measure in the input space. Mathematically speaking, metric tensor induces in its diagonal elements feature scaling, while also accounting for different interplay between pairs of features (co-ordinates of the input space).

Given a positive definite matrix $\Lambda$, $\Lambda\succ0$, the generalised form of the squared distance is calculated as

$d_{\Lambda}\left( x,w \right)={(x-w)}^{T}\Lambda(x-w)$.

Positive definiteness of $\Lambda$ can be ensured by defining $\Lambda=Ω^{T}Ω$ where $Ω \in\mathbb{R}^{m \times m}$ is a full rank matrix. Note that only relative distances of input points to the prototypes are important. Hence, the metric tensor can be multiplied by any positive real number without effecting the classifier performance in any way. To account for this inherently ill-posed nature of the model fitting and hence to ensure the stability of the algorithm, $\Lambda$ must be normalised following each learning step e.g. by making sure that $\sum_{i} \Lambda_{i,i}=1$, fixing the trace throughout learning.

Using the steepest descent method, the cost function to be minimised through online learning is

$$f_{\mathrm{GMLVQ}}=\sum_{i=1}^{n} \varphi\left( \mu_{\Lambda}(x_{i}) \right)$$

where

$$\mu_{\Lambda}\left( x_{i} \right)=\frac{d_{\Lambda}\left( x_{i},w^{+} \right)-d_{\Lambda}\left( x_{i},w^{-} \right)}{d_{\Lambda}\left( x_{i},w^{+} \right)+d_{\Lambda}\left( x_{i},w^{-} \right)}$$

In this instance $\varphi$ is a monotonic identity function$\varphi\left( \mathcal{l} \right)\mathcal{=l}$, $d_{\Lambda}\left( x_{i},w^{+} \right)$ is the distance between the sample vector $x_{i}$ from the closest prototype with the same class label ${c(w)}^{+}={c(x}_{i})$ and $d_{\Lambda}\left( x_{i},w^{-} \right)$is the distance from $x_{i}$ to the closest prototype of a different class. We assessed model performance by classification accuracy, true positive rate, true negative rate and macro averaged error (MAE).

**Figure S1: Interrogating the Metric Tensor Matrix**

Figure S1 illustrates 3 different scenarios where scaling the original data by the metric tensor has ensured that all points have a neighbour of the same class i.e. the shortest pairwise distance for all points is with a point from the same class.

S1c-iiii

S1c-iii

S1b-iiii

S1b-iii

S1a-iiii

S1a-iii

S1c-i

S1c-ii

S1b-ii

S1b-i

S1a-ii

S1a-i


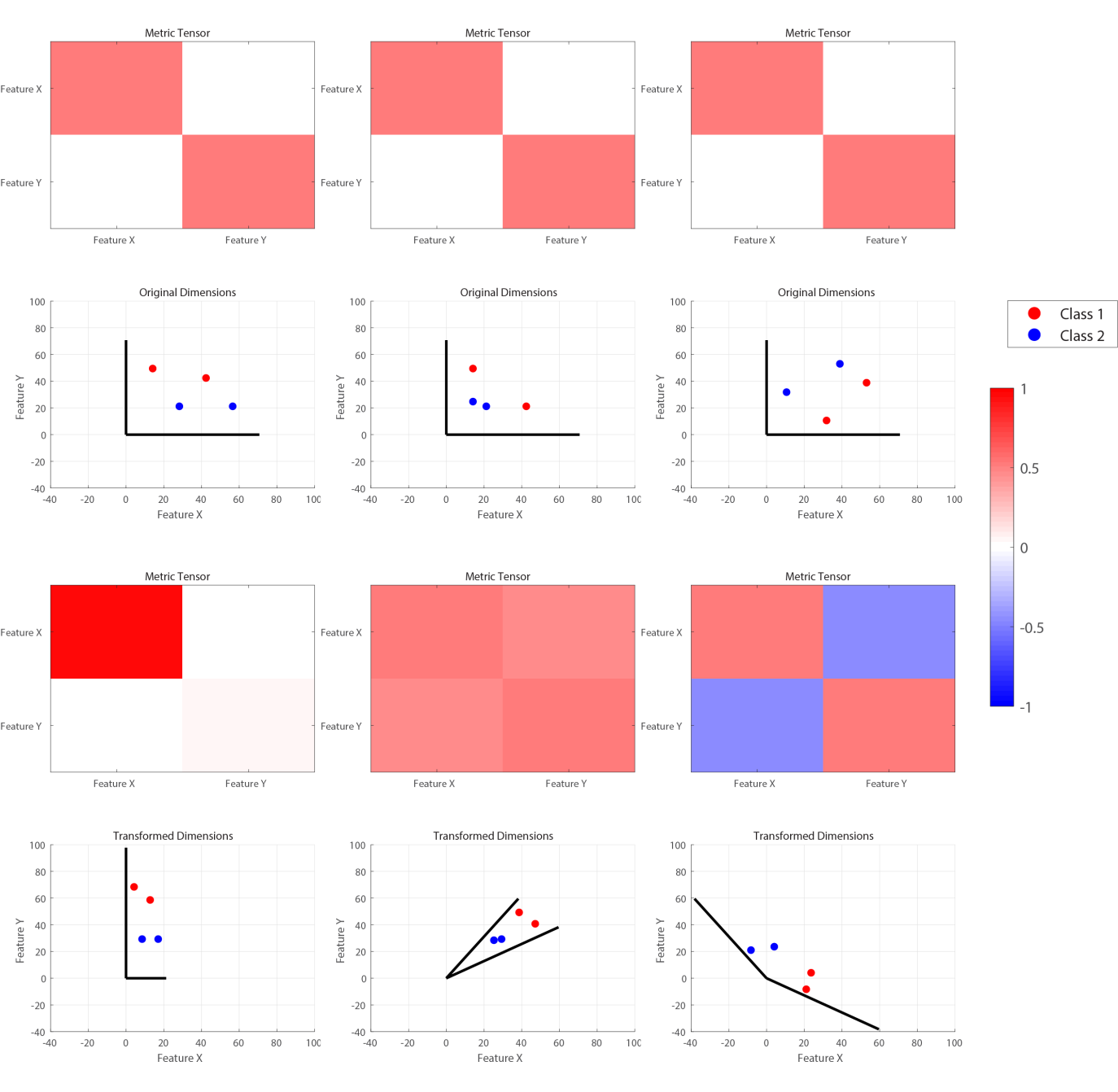


*Figures S1a-i – S1c-i shows the initialised metric tensor. Figures S1a-ii – S1c-ii shows three different examples of data distribution in the original space, where the red circles are training individual data from class 1 and the blue circles are training individual data from class 2. The solid black line represents the X and Y axis in the original space. Figures S1a-iii – S1c-iii show the learnt metric tensor following the GMLVQ process for the three different examples; note the diagonal terms sum to 1 and the off diagonals can be either negative of positive. Figures S1a-iiii – S1c-iiii show the transformed space when scaled by the learnt metric tensor, the red circles are the same individuals from Figures S1a-ii – S1c-ii in the transformed space following the GMLVQ process and the solid black lines show the transformation of the X and Y axis into the learnt space.*

Panel S1a (left) shows that all data have a neighbour of the same class the diagonals need to be scaled so that the term for Feature X has to be increased relative to Feature Y. The conclusion that is drawn from the metric tensor is that Feature X is the most relevant feature. In panel S1b (middle) we show that for all data to have a neighbour of the same class the interaction between Feature X and Feature Y is highly relevant (Note that this term is positive). The positive term collapses the angle between the two dimensions causing data of the same class to group together. Panel S1c(right) shows that for all data to have a neighbour of the same class the interaction between Feature X and Feature Y is relevant; however as this term is negative it increases the angle between the two dimensions. Thus,we conclude that these two features separate points from different classes.

To interpret figure S1 mathematically, note that the squared distance can be written as

$d_{\Lambda}\left( x,w \right)=\left( x-w \right)^{T}\Lambda\left( x-w \right)= \left( X-W \right)^{T}\left( X-W \right)= d_{2}\left( X,W \right)$,

where $d_{2}\left( .,. \right)$ denotes the squared Euclidean distance and ${X=\Lambda}^{1/2}x$, ${W=\Lambda}^{1/2}w$. Hence, the original vectors (including the unit directional vectors of the standard axis) are transformed by the linear operator equal to the square root of the symmetric positive-definite metric tensor $\Lambda$ into a space where the new distance (defined by $\Lambda$) can be re-interpreted as the usual Euclidean distance.

***Methods GMLVQ – Scalar Projection***

Following the learning process in GMLVQ we transformed the sample vector $x_{i}$ and prototypes $w_{(stable,progressive)}$into the learnt space via the metric tensor$\Lambda$.

$$X_{i}=\Lambda^{1/2} x_{i}$$

$$W_{(stable,progressive)}=\Lambda^{1/2}w_{(stable,progressive)}$$

As the metric tensor $\Lambda$ is learnt in the non-Euclidean space:$d_{\Lambda}\left( x,w \right)={(x-w)}^{T}\Lambda(x-w)$, we applied the square root of this tensor to re-represent the data so that the squared norm of a vector in the non-Euclidian space is equal to the squared Euclidean norm of the transformed space.

We centred the coordinate system on $W_{(stable)}$ and calculated the orthogonal projection of each vector $X_{i}$ onto the vector $W_{\left( progressive \right)}$, in this co-ordinate system.

$$Projection=\frac{X_{i}W_{stable}.W_{progressive}W_{stable}}{\left| W_{progressive}W_{stable} \right|}$$

To normalise the projections with respect to the position of the prototype $W_{(progressive)}$, the we divided the projection by the norm of $W_{(progressive)}$:

$$Scalar Projection=\frac{X_{i}W_{stable}.W_{progressive}W_{stable}}{\left| W_{progressive}W_{stable} \right|^{2}}$$

The resultant value indicates the separation of a test point from prototype $W_{stable}$ along the direction of $W_{progressive}W_{stable}$. To determine the relative separation from the stable prototype, we normalised the projection by the distance between each prototype $W_{progressive},W_{stable}$, as indicated by squaring the norm of vector $W_{progressive}W_{stable}$. A large positive direction indicates a large separation from $W_{stable}$ in direction $W_{progressive}W_{stable}$ and a large negative projection indicates a large separation from $W_{stable}$in the opposite direction i.e. $W_{stable}W_{progressive}$. A value of 1 indicates that a sample is incident to prototype $W_{progressive},$whereas a value of 0 indicates that a sample is incident to prototype $W_{stable}$, and a value of 0.5 is the decision boundary separating the two classes within the binary classification framework. The scalar projection has a large positive value for pMCI individuals and zero or negative value for sMCI individuals.

***Figure S2: Deriving the GMLVQ – Scalar Projection: Prototype Vector***

Figure S2 uses the example in figure S1b to graphically derive the Prototype Vector ($W_{j}W_{q})$ that is generated following the GMLVQ learning process.


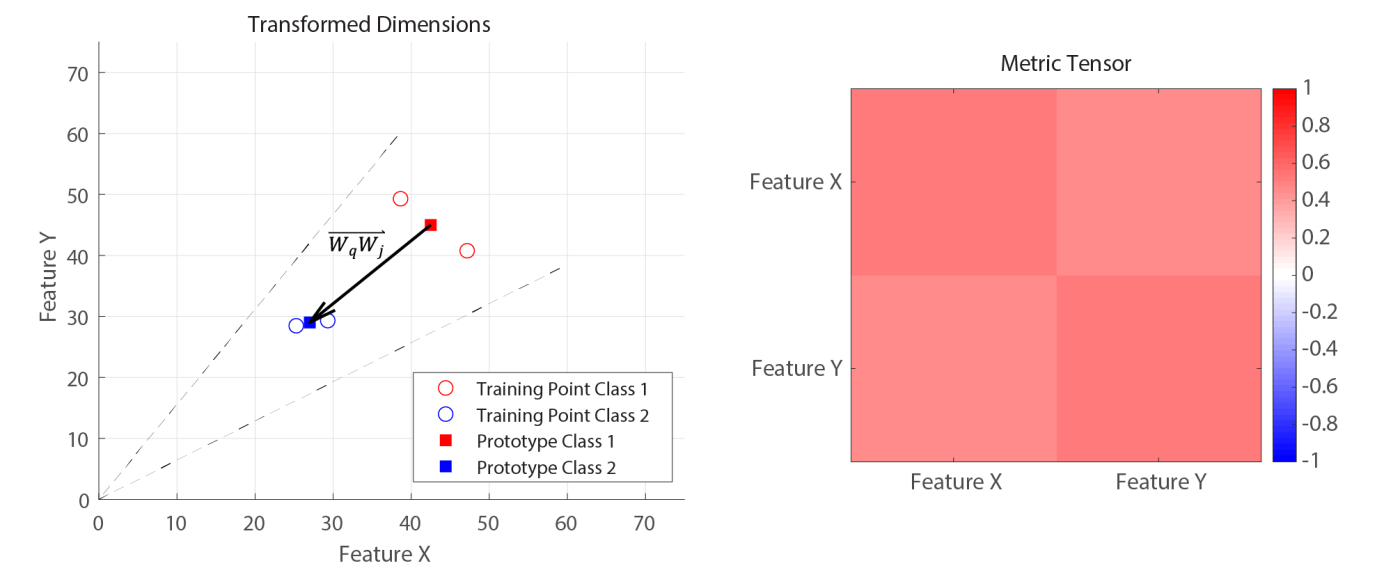
*Figure S2a shows the vector* $W_{j}W_{q}$ *represented by the black arrow connecting prototypes for class 1 and class 2. The dashed lines show the transformed axis from the original space. Figure S2b is the learnt metric tensor used to transform the data from the original space into the learnt space.*

***FigureS3: Deriving the GMLVQ – Scalar Projection: Test Projections***

Figure S3 shows the graphical derivation of the scalar projection for 3 different test points from the model used in figures S1b and S2.

S3c-i

S3b-i

S3a-i


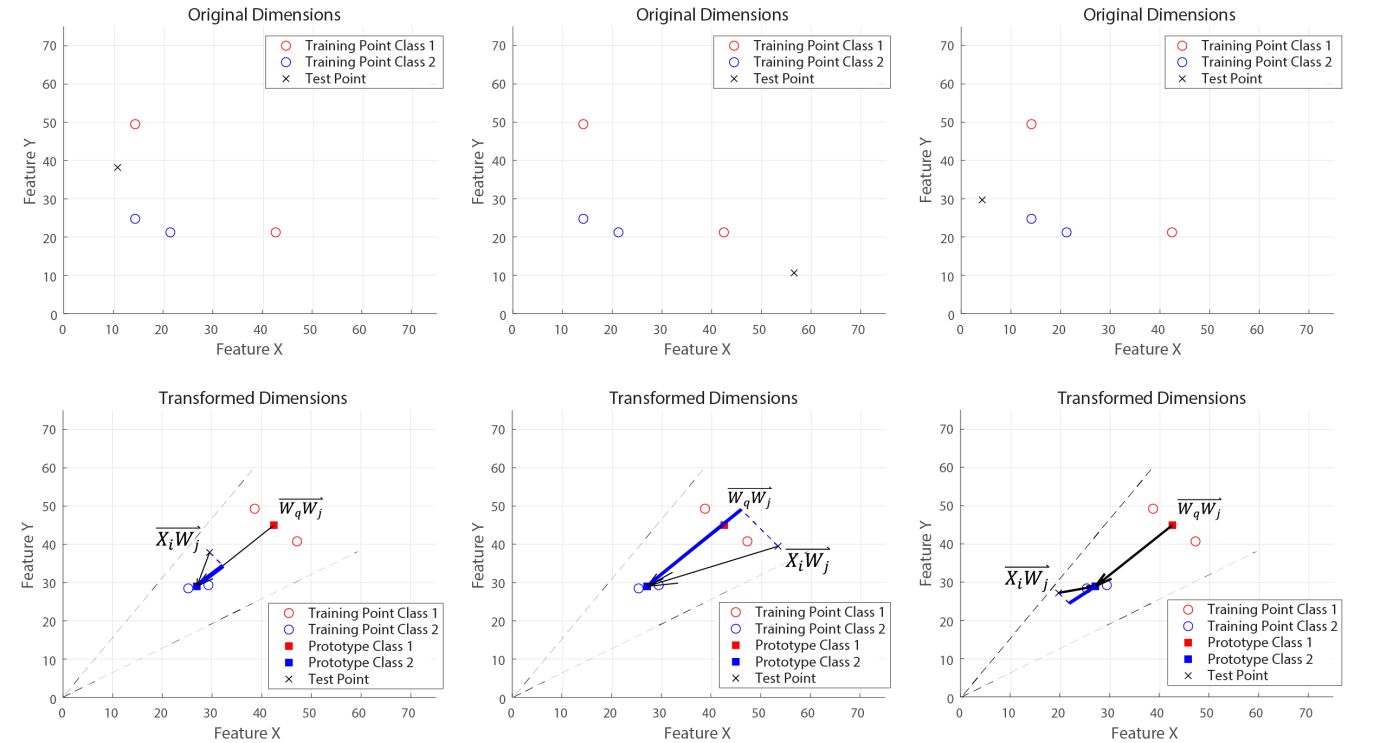


S3c-ii

S3b-ii

S3a-ii

*Figures S3a – S3ci show the three different test points in the original data space. Figures S3aii – S3cii shows three test points from S3ai-ci and the scalar projections in the learnt space. The vector* $W_{j}W_{q}$ *connects the prototypes for class 1 and class 2, vector* $X_{i}W_{q}$ *connects the test point in the learnt space with the prototype for class 2. The dashed blue line is the orthogonal projection from the test point in the learnt space to vector* $W_{j}W_{q}$*. The solid blue line represents the scalar projection from the test point in the learnt space to the class 2 prototype along the vector* $W_{j}W_{q}$*.*

The three scenarios shown in figures S3a – S3c give an example of three different values for the scalar projection. Figure S3a shows an example of a scalar projection that has a length between 0 and 1. Within the binary classification framework a decision boundary exists at 0.5, where a value below this will cause the point to be labelled as class 2 and a value beyond this will cause the point to be labelled class 1. This is interpreted as an individual that is between the two classes. In figure S3b an example is given where the length of the scalar projection is larger than 1, in this example the individual is further away from class 2 prototype along the prototype vector than the class 1 prototype. This is interpreted as being further away from the class 2 prototype than the majority of class 1 individuals. Figure S3c shows an example where the length of the scalar projection is negative. In this example the individual is further away from the class 1 prototype than the class 2 prototype along the prototype vector. This is interpreted as being further away from the class 1 prototype than the majority of class 2 individuals.

***Methods Cross Validation Framework***

**Within-sample validation: *k-fold cross-validation (GMLVQ/PLSr-RFE)***

Within-sample generalisation for the GMLVQ (Development Sample (b)) and PLSr-RFE (Development Sample) frameworks was assessed using k -fold cross validation (GMLVQ k=10, PLSr-RFE k=5). In brief, data was split into a series of training and test sets, whereby each sample point is present once in a test set. To select hyper-parameters for a given training sample, we used nested cross-validation. Within each fold, we used grid search in the parameter space; that is, we folded our training data into hyper parameter cross-validation test and training sets and searched across all possible combinations of hyper parameters. We selected the optimal set of hyper parameters for a given cross fold based on mean performance across hyper parameter cross-validation test sets. We then fixed the hyper parameters within each cross fold and train the models. To assess model generalisation performance, we averaged metrics (GMLVQ: Accuracy, Macro Averaged Error (MAE), True Positive (TP), True Negative (TN); PLSr-RFE: Variance Explained) from the test set across all cross folds.

***Resampling GMLVQ***

We performed a resampling throughout the GMLVQ learning procedure to address class imbalance for the binary classification tasks. That is, within each cross fold, we down-sampled the majority class to the same number of samples as the minority class. We performed a random down-sampling of the data set 100 times respecting the class split defined, thus creating 100 classifiers forming an ensemble. To determine the class of a given test point we performed majority voting among the 100 classifiers in the ensemble. Similarly, we interrogated the efficacy of our selected variables to perform the classification task via the average metric tensor across all resamples and cross folds. Note that in our case, it is possible to use the average metric tensor, as the variability in the population of metric tensors across resamples and folds is small.

***Resampling PLSr-RFE***

To generate our test cross-folds and validation cross-folds, we performed a stratified permutation. As we have a priori knowledge of the distribution of the ADNI-Mem score within our complete sample, we respected this distribution within each fold of the data. To do this we ordered our sample by ADNI-Mem and resampled the data to have population representative distributions relative to this metric.

**Within-sample validation: *Random resampling (GMLVQ-Scalar Projection)***

Within-sample generalisation for the GMLVQ-scalar projection framework (Development Sample I) was assessed using random resampling. In brief, we tested the relationship between the scalar projection and rates of future cognitive decline by randomly splitting our sample into test and training data 1000 times. To avoid biasing the model in the training phase due to class imbalance in the data (majority class: sMCI= 113 vs. minority class: pMCI = 54), we resampled the data to generate balanced classes (i.e. number of sMCI equals number of pMCI individuals). This resampling process randomly selects half of the individuals in the minority class and the same number of individuals from the majority class as training data; with the remaining sample used as test data. The training data within a resampling was used in the GMLVQ framework to learn the metric tensor and prototype locations for each training set. We then calculated the GMLVQ-Scalar Projection for the corresponding test set and correlated these values (Pearson’s correlation) with the rate of future ADNI-Mem change for the same individuals. We assessed within-sample generalisation based on the median of the correlation coefficients generated from the test sets across resampling using 95% confidence intervals.

***Out-of-Sample validation***: ***Cross-modal associations (PLSr-RFE)***

To test the out-of-sample association of the PLS derived grey matter feature (represented by the voxel weight matrix) with memory and cortical tau we used an independent sample of individuals from ADNI 3 (Cross modal associations validation sample I). We pre-processed the structural MRI scans independent of the Development sample used for model formulation following the same VBM methodology. We derived a grey matter score for the validation sample by multiplying the weight matrix generated from the PLSr-RFE trained on Development sample by the grey matter voxel values of the Cross-modal associations validation sample. We then correlated the grey matter score from the Cross-modal associations validation sample I with the ADNI-Mem composite score to test the generalisability of the grey matter score across data samples. Further, we tested the cross modal association of the PLS derived grey matter score with cortical tau burden (measured with FTP-PET) (Cross-modal associations validation sample). Using independent sample t-tests, we tested whether there is a significant difference in PLS derived grey matter score between tau positive and tau negative individuals across 3 different Braak staging regions: 12, 34 and 56. We then tested whether individual variability in the PLS derived grey matter score relates to individual variability in cortical tau burden across each of the Braak staging regions (Pearson’s Correlation).

***Out-of-Sample validation***: ***Longitudinal Predictions (GMLVQ-Scalar Projection)***

To test the out-of-sample generalisability of the GMLVQ-Scalar Projection in predicting individual rates of future cognitive decline, we drew a validation sample with longitudinal information (i.e. cognitive data from three or more measurements) (Longitudinal prediction validation sample). As described above, we derived the grey matter score for this validation sample from the voxel weight matrix generated from the PLSr-RFE using the Development Sample. To generate the scalar projections for the longitudinal validation sample, we used the metric tensor and prototype locations generated from the trained model with the median test performance on Development sample II. Using the metric tensor and prototype locations from the cognitive and biological models, we then generated a cognitive scalar projection and a biological scalar projection for each individual in the longitudinal validation sample. We then correlated these scalar projections with the rate of change in future ADNI-Mem scores (i.e. following baseline). Correlation coefficients were computed using skipped Pearson Correlation (Robust Correlation Toolbox; (Pernet et al., 2013). This method accounts for potential outliers and determines significance using bootstrapped confidence intervals (CI) from 1000 permutations.

***Comparing Correlations between samples***

To test if the relationship between the GMLVQ-Scalar Projection and rate of future cognitive decline is significantly different between Development sample II and the Longitudinal prediction validation samples, we used Fisher’s r to Z transformation. The Fisher’s Z statistic allows us to compare correlations from independent samples. To compare if the relationship of the GMLVQ-Scalar Projection and rate of future cognitive decline is significantly different between models using biological vs. cognitive data, we generated a Steiger Z statistic (Steiger, 1980). The Steiger Z statistic allows us to compare correlations between data with a shared dependent variable (i.e. rate of memory change).

**Supplementary Results**

**Composite grey matter score for predicting cross-modality associations**

**Nested Cross Validation Results**

**Optimising Number of Remaining Voxels**

To determine the optimal number of voxels to retain in the PLSr-RFE model, we used a nested cross validation framework. Our acceptance criterion is the amount of variance explained in the ADNI-Mem score from the cross validation hold out set. We folded our training set 5 times to tune the parameter of number of retained voxels**.** Further, we investigated how the PLSr-RFE model performs when the same grey matter data is modulated in the VBM process.

**PLS 1 on un-modulated grey matter:** For the first PLS dimension across cross validation runs we observed a decline in test variance explained after the 21st recursive loop iteration corresponding to 902 remaining voxels. Figure S4 shows the converging performance of the training and test sets throughout the recursive elimination process. Here, we show that generalisation performance approximately doubles when eliminating variant voxels.

***Figure S4a: PLS 1* on un-modulated grey matter *Early Stopping Generalisation Performance***


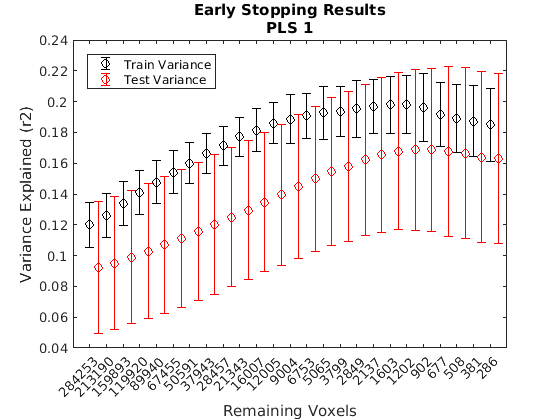


*Figure S4a shows the training and test performance of PLS 1 across each recursive elimination iteration using un modulated grey matter data. The black dots and error bars show the mean and standard deviation of the variance explained in the ADNI-Mem score by PLS1 for the training sample across all nested validation loops. The red* *dots and error bars show the mean and standard deviation of the variance explained in the ADNI-Mem score by PLS1 for the test sample across all nested validation loops.*

**PLS 1 on modulated grey matter:** For the first PLS dimension across cross validation runs we observed a decline in test variance explained after the 16th recursive loop iteration corresponding to 3799 remaining voxels. Figure S4b shows a different profile to that of the un-modulated data (Figure S4a) on the training and test sets throughout the recursive elimination process. Here, we show that generalisation performance peaks with approximately 4 times the amount of voxels retained than the un-modulated data.

***Figure S4b: PLS 1*on modulated grey matter *Early Stopping Generalisation Performance***

*
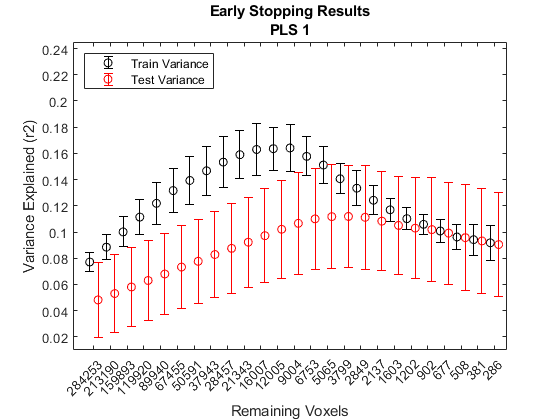
*

**Generalisation Performance:**

To determine the efficacy of the PLS derived voxel weights matrix to generalise to another sample, we performed the matrix multiplication of the hold-out grey matter and the voxel weights matrix within each cross fold following the recursive elimination process. Table S2a shows the hold out performance for each of the five cross folds using un-modulated data. The high correspondence between the training and test variance across the cross folds suggests that the PLS derived voxel weights matrix generated is robust and specific to the cognitive measure ADNI-Mem.

**Table S2a: PLS 1 Cross Fold Performance**

|  | **train variance** | **test variance** |
| --- | --- | --- |
| **cross fold** | **MEM** | **MEM** |
| 1 | 0.16 | 0.28 |
| 2 | 0.18 | 0.18 |
| 3 | 0.18 | 0.18 |
| 4 | 0.21 | 0.09 |
| 5 | 0.20 | 0.14 |
| MEAN | 0.19 | 0.18 |

*Table S2a shows the training and test variance explained in the ADNI Mem score by PLS derived grey matter score for the un-modulated data from the individual cross folds and the mean performance across all cross folds.*

**Table S2b: PLS 1 Cross Fold Performance**

|  | **train variance** | **test variance** |
| --- | --- | --- |
| **cross fold** | **MEM** | **MEM** |
| 1 | 0.15 | 0.07 |
| 2 | 0.13 | 0.05 |
| 3 | 0.13 | 0.05 |
| 4 | 0.13 | 0.06 |
| 5 | 0.12 | 0.04 |
| MEAN | 0.13 | 0.05 |

*Table S2b shows the training and test variance explained in the ADNI Mem score by PLS derived grey matter score for the modulated data from the individual cross folds and the mean performance across all cross folds.*

**Spatial Organisation of Voxel Weights Matrix**

Table S3 lists the z-statistics for each cluster contained within a given anatomical region within the average voxel weights matrix across cross folds. We show that the voxels cluster within the medial temporal cortex covering regions in the bi-lateral hippocampus and amygdala. Reference regions of interest (ROIs) are extracted from the Brainnetome Atlas (Fan et al., 2016).

**Table S3: PLS 1**

| **%Overlap** | **Max Z** | **Mean Z** | **nVoxels** | **ROI (Left/Right)** | **Anatomical** **Description (BA Area)** |
| --- | --- | --- | --- | --- | --- |
| 0.55 | 9.8 | 9.08 | 13 | FuG L | *rostroventral (area 20)* |
| 6.2 | 10.29 | 9.67 | 24 | PhG L | *rostral (area 35/36)* |
| 17.2 | 15.22 | 11.76 | 66 | Amyg L | *medial amygdala* |
| 18.3 | 15.2 | 11.89 | 90 | Amyg R | *medial amygdala* |
| 21.29 | 16.44 | 12.71 | 43 | Amyg L | *lateral amygdala* |
| 14.29 | 15.7 | 13.09 | 45 | Amyg R | *lateral amygdala* |
| 18.28 | 17.69 | 12.49 | 247 | Hipp L | *rostral hippocampus* |
| 6.59 | 13.87 | 11.59 | 73 | Hipp R | *rostral hippocampus* |
| 6 | 14.64 | 11.88 | 82 | Hipp L | *caudal hippocampus* |
| 10.09 | 12.81 | 10.88 | 144 | Hipp R | *caudal hippocampus* |

*Table S3 reports brain regions within the mean PLS 1 voxel weights matrix across cross folds. The table shows the percentage overlap of each ROI with the voxel weights matrix, the peak Z value within each region, the average Z value within each region, the number of voxels and the gross and fine anatomical description of the regions. FuG: Fussiform Gyrus, PhG: Parrahippocampal Gyrus, Amyg: Amygdala, Hipp: Hippocampus, L:Left, R:Right*

**PLS derived grey matter score vs. A-Priori Regions**

We compared the variance explained in ADNI-Mem by the PLS derived grey matter score with the mean grey matter density from a set of corresponding regions known to be related to ADNI-Mem (Nho et al., 2012). For each of the test sets in our nested cross validation framework, we derived the PLS grey matter score and extracted the mean grey matter density from 8 anatomically defined ROIs (left/right medial Amygdala, left/right lateral Amygdala, left/right rostral hippocampus, left/right caudal hippocampus) taken from the Brainnetome Atlas (Fan et al., 2016). Further, we extracted the mean grey matter density across all 8 medial temporal regions (**table S4)**. We found that the variance explained in the hold out test set for the PLSr-RFE framework (i.e. generalisation variance) was significantly higher than the variance explained by grey mater density from the a-priori selected regions.

**Table S4:** **Generalisation variance explained: A-Priori Regions vs. PLS derived grey matter score**

| **Region** | ***Anatomical Description*** | **L** | | **R** | |
| --- | --- | --- | --- | --- | --- |
|  |  | **p** | **Cohens D** | **p** | **Cohens D** |
| Amygdala | *mAmyg, medial amygdala* | **<.0001** | **-1.14** | **0.01** | **-0.55** |
| Amygdala | *lAmyg, lateral amygdala* | **<.0001** | **-1.06** | **<.0001** | **-1.05** |
| Hippocampus | *rHipp, rostral hippocampus* | **<.0001** | **-1.31** | **<.0001** | **-1.21** |
| Hippocampus | *cHipp, caudal hippocampus* | **<.0001** | **-2.02** | **<.0001** | **-1.09** |
| **Aggregate Region** | **Aggregate Description** | **p** | | **Cohens D** | |
| Mean | Average Medial Temporal Regions | **<.0001** | | **-1.12** | |

*Table S4 shows the statistics and effect sizes when comparing the amount of variance explained in the ADNI Mem score by the a-priori selected areas and the PLS derived grey matter score across all nested cross folds.*

**Validation of scalar projection: Future rate of ADNI-Mem change with incomplete (missing) data.**

We next asked whether the GMLVQ-scalar projection approach could be implemented to predict future rate of cognitive decline for data with less than 3 years of clinical diagnosis. To incorporate these incomplete data from the model trained on Development Sample II, we chose the metric tensor and prototype positions from the model with the median test performance. We observed that scalar projections from both models (cognitive and biological) account for significant variance in the rate of future memory decline when the incomplete data was included in the test set (Note that data used to train the model with median test performance (n=52) were not used to test the relationship between the scalar projection and rates of future cognitive decline) (Figure S5). For the test sample used for the Cognitive model (Development Sample I) we observed a significant correlation between the cognitive scalar projection and the rate of future cognitive decline: [r(196) = -0.4 (95% CI: [-0.51 -0.29]), P < 0.0001] that remained significant when including only incomplete data ([r(85) = -0.31 (95% CI: [-0.48 -0.13]), P < 0.0001]). For the test sample used for the Biological model (Development Sample I) we observed a significant correlation between the biological scalar projection and the rates of future cognitive decline [r(196) =-0.51 (95% CI:[-0.6 -0.4]), P < 0.0001], that remained significant when including only incomplete data ([r(85) = (-0.45 (95% CI: [-0.59 -0.3]), P < 0.0001]). The observed r values for models incorporating incomplete data were comparable to the correlation values between the scalar projection and the rate of cognitive decline for individuals with complete clinical longitudinal information (Development Sample II: 3 years of clinical assessment) (Fisher’s r to Z, Cognitive: [Z=-0.86, P=0.39], Biological: [Z=-1, P=0.32]). These findings suggest that the GMLVQ-scalar projection approach allows us to make longitudinal predictions based on the rate of future cognitive decline from a limited number of longitudinal clinical assessments (i.e. less than 3 years).

**Figure S5: Correlating GMLVQ-Scalar projections with rate of memory change:**


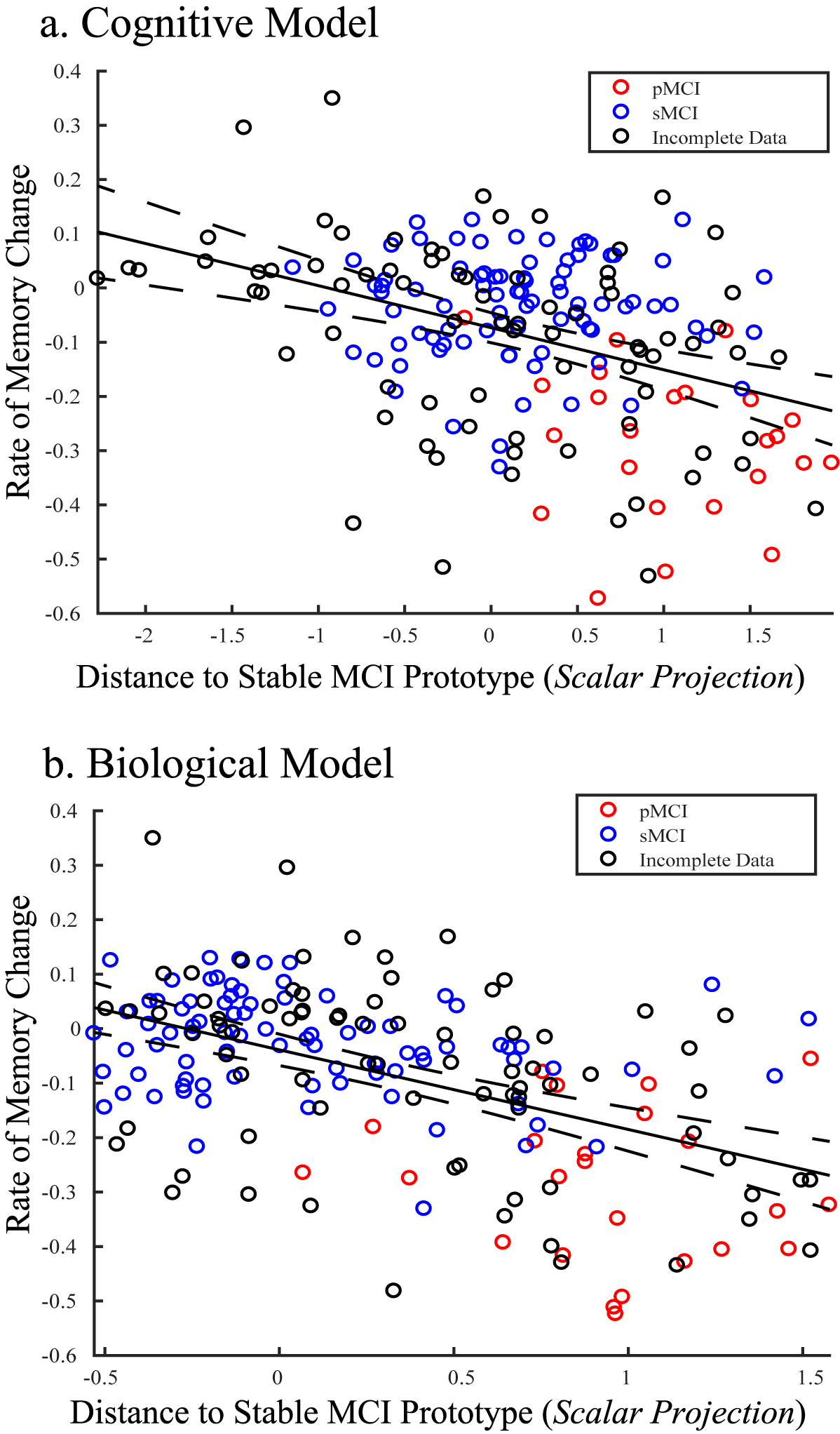


Figure S5 shows the correlation of the GMLVQ-scalar projections derived from the a) cognitive model, b) biological model with the rate of ADNI-Mem change for Development Sample I. Red dots indicate pMCI individuals, blue dots indicate sMCI individuals and black dots indicate individuals with incomplete data. The central black line is the regression line for the fit of the GMLVQ-scalar projection to the rate of ADNI-Mem change; the dashed lines represent the 95% confidence intervals for this regression line. Data used to train the model (n=52) were not used to test the relationship between the scalar projection and rates of future cognitive decline and are not shown. Outliers identified by the Robust Correlation toolbox (cognitive: 11, biological=11) are not shown.

**Validation of scalar projection: Future rate of MMSE change**

To further validate the clinical relevance of our scalar projection approach, we tested whether the scalar projections derived from either the cognitive or biological model relate to the rate of future MMSE change, as MMSE is typically used in clinical diagnosis. We observed that the scalar projections correlated significantly with the future rate of change in the MMSE score (Cognitive: [r(214) = -0.53, P < 0.0001], Biological: [r(213) = -0.43, P <0.0001] (Figure S6).

**Figure S6: Correlating Scalar projections from Cognitive and Biological Models with rate of MMSE change**


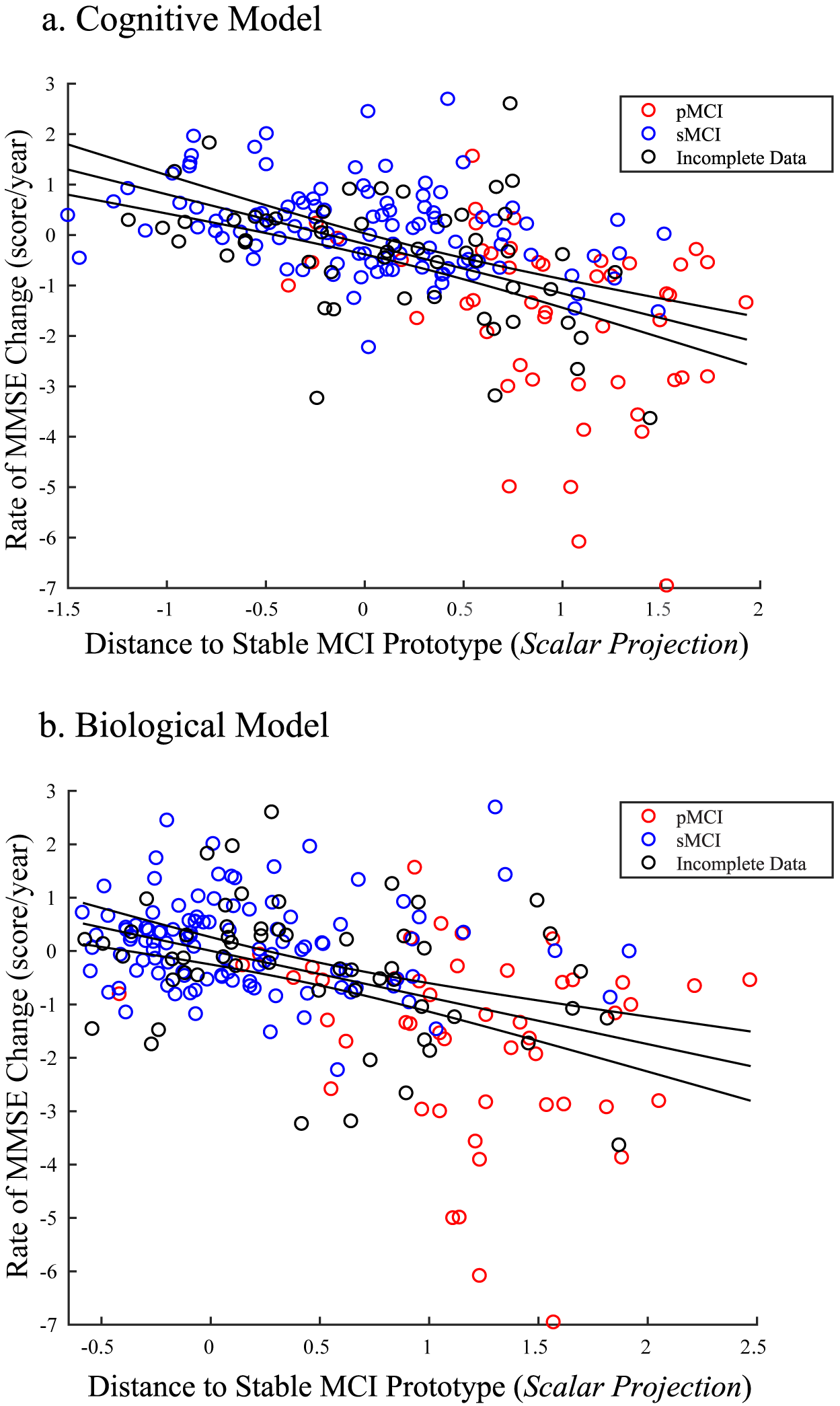


Figure S6 shows the correlation of scalar projection derived from the a) cognitive model, b) biological model with the rate of MMSE change for the original data sample. The central black line is the regression line for the fit of the GMLVQ-scalar projection to the rate of MMSE change; the dashed lines represent the 95% confidence intervals for this regression line. Red circles indicate pMCI individuals, blue circles sMCI individuals and black dots for the incomplete data (i.e. less than three years of clinical assessments). Note: 23 Individuals from the previous analysis had fewer than 3 MMSE tests from baseline.

***References:***

Fan, L., Li, H., Zhuo, J., Zhang, Y., Wang, J., Chen, L., Yang, Z., Chu, C., Xie, S., Laird, A.R., Fox, P.T., Eickhoff, S.B., Yu, C., Jiang, T., 2016. The Human Brainnetome Atlas: A New Brain Atlas Based on Connectional Architecture. Cereb. Cortex 26, 3508–3526. https://doi.org/10.1093/cercor/bhw157

Krishnan, A., Williams, L.J., McIntosh, A.R., Abdi, H., 2011. Partial Least Squares (PLS) methods for neuroimaging: A tutorial and review. Neuroimage 56, 455–475. https://doi.org/10.1016/J.NEUROIMAGE.2010.07.034

McIntosh, A.R., Lobaugh, N.J., 2004. Partial least squares analysis of neuroimaging data: Applications and advances. Neuroimage 23, 250–263. https://doi.org/10.1016/j.neuroimage.2004.07.020

Milan, L., Whittaker, J., 1995. Application of the Parametric Bootstrap to Models that Incorporate a Singular Value Decomposition. Appl. Stat. 44, 31. https://doi.org/10.2307/2986193

Nho, K., Risacher, S.L., Crane, P.K., DeCarli, C., Glymour, M.M., Habeck, C., Kim, S., Lee, G.J., Mormino, E., Mukherjee, S., Shen, L., West, J.D., Saykin, A.J., 2012. Voxel and surface-based topography of memory and executive deficits in mild cognitive impairment and Alzheimer’s disease. Brain Imaging Behav. 6, 551–567. https://doi.org/10.1007/s11682-012-9203-2

Pernet, C.R., Wilcox, R., Rousselet, G.A., 2013. Robust Correlation Analyses: False Positive and Power Validation Using a New Open Source Matlab Toolbox. Front. Psychol. 3, 606. https://doi.org/10.3389/fpsyg.2012.00606

Schneider, P., Biehl, M., Hammer, B., 2009. Adaptive Relevance Matrices in Learning Vector Quantization. Neural Comput. 21, 3532–3561. https://doi.org/10.1162/neco.2009.11-08-908

Steiger, J.H., 1980. Tests for comparing elements of a correlation matrix. Psychol. Bull. 87, 245–251. https://doi.org/10.1037/0033-2909.87.2.245
